# Extracellular Vesicles Administered via Intrathecal Injection Mediate Safe Delivery of Nucleic Acids to the Central Nervous System for Gene Therapy

**DOI:** 10.1002/jev2.70116

**Published:** 2025-07-07

**Authors:** Brenda Wan Shing Lam, Melissa Tan, Chang Gao, Thach Tuan Pham, Lan Thi Ngoc Tran, Lan Ngoc Nguyen, Harwin Sidik, Matthias B. H. Lim, Anh Hong Le, Tram T. T. Nguyen, Nicole Lim, Lap Nguyen, Thuy Huong Nguyen, Yuin‐Han Loh, Muhammad Waqas Usman, Minh T. N. Le

**Affiliations:** ^1^ Department of Pharmacology Yong Loo Lin School of Medicine National University of Singapore Singapore; ^2^ Institute for Digital Medicine Yong Loo Lin School of Medicine National University of Singapore Singapore; ^3^ Carmine Therapeutics Singapore; ^4^ Department of Biochemistry Hanoi University of Pharmacy Hanoi Vietnam; ^5^ Center for Research and Production of Vaccines and Biologicals Hanoi Vietnam; ^6^ Agency for Science, Technology and Research Institute of Molecular and Cell Biology Singapore; ^7^ Department of Surgery Yong Loo Lin School of Medicine National University of Singapore Singapore

**Keywords:** central nervous system, extracellular vesicles, gene therapy, non‐human primates, plasmid delivery, safety

## Abstract

Gene therapy holds great potential for treating neurological disorders, but its implementation is limited by the challenge of developing a safe and effective delivery method to the central nervous system (CNS). Red blood cell‐derived extracellular vesicles (RBCEVs) have the potential to address these challenges due to their non‐immunogenicity, non‐cytotoxicity, ability to be redosed, and suitability for nucleic acid loading. In this study, we demonstrate the efficacy and safety of RBCEV‐mediated nucleic acid delivery to the CNS. We found that RBCEVs administered through intrathecal injection are widely distributed across the CNS and efficiently taken up by neuronal cells. Delivery of RBCEVs loaded with GFP‐encoding plasmids results in GFP expression in neurons. Our data also highlight the potential of RBCEVs to deliver plasmids encoding secretory proteins, resulting in protein secretion within the cerebrospinal fluid. Furthermore, experiments conducted in both mouse and non‐human primate models indicate that intrathecal injection of plasmid‐loaded RBCEVs do not lead to any systemic or local acute toxicity. In summary, our findings illustrate the potential of the RBCEV‐based platform as a viable and safe approach for nucleic acid delivery to the CNS, facilitating further development of gene therapy for neurological disorders.

## 1 Introduction

The central nervous system (CNS) plays an important role in receiving, processing and responding to sensory signals to ensure one's survival and well‐being (Rouleau et al. 2023). It comprises of the brain and spinal cord, which regulates various physiological functions including cognition, motor control and sensory perception (Rouleau et al. 2023). Given its significance in these functions, any condition or disorder that impacts the CNS can detrimentally affect a person's quality of life and may even lead to death if left untreated. Consequently, advancing therapeutic approaches to target CNS disorders is important.

Among the different treatment modalities available, nucleic acid‐based treatments are distinguished by their potential to produce long‐lasting or even permanent outcomes (Kulkarni et al. 2021; Wang et al. 2023). Gene therapy involves directly modifying genes or regulating gene expression. This is in contrast with protein‐based therapies, which frequently yield short‐term effects and do not address the underlying genetic issues (Kulkarni et al. 2021; Wang et al. 2023). Unfortunately, many gene therapy strategies face constraints in their applications due to limitations of the currently available delivery systems (Kulkarni et al. 2021; Wang et al. 2023).

Effective gene delivery requires the safe and efficient transfer of genetic material into specific cells, a challenge that is particularly pronounced in the context of the CNS. The CNS is a difficult site to access for many nano‐sized particles, largely due to the blood‐brain barrier (BBB). Systemic delivery methods, such as intravenous injections, require substantially higher doses, but achieve minimal efficacy in the CNS (Wu et al. 2023). Traditional delivery vehicles such as lipid nanoparticles (LNPs) and adeno‐associated viruses (AAVs) have demonstrated high transfection efficiencies in vitro (Barua and Mitragotri 2014; Bulcha et al. 2021; Hargrove et al. 2008; Kuranda et al. 2018; Lee et al. 2023; Maes et al. 2019; Nisini et al. 2018). However, systemic delivery using these modalities is often hindered by toxicity, immunogenicity, limited payload capacity and variable effectiveness, especially in the delivery of DNA plasmids (Barua and Mitragotri 2014; Bulcha et al. 2021; Hargrove et al. 2008; Kuranda et al. 2018; Lee et al. 2023; Maes et al. 2019; Nisini et al. 2018). In addition, their limited ability to extravasate across endothelial and perivascular barriers, often leads to their accumulation in clearance organs such as the liver and spleen (Barua and Mitragotri 2014). This restricted biodistribution limits their effectiveness in treating CNS diseases.

Local drug administration can help overcome these challenges by bypassing the BBB, enabling lower dosing regimens while increasing local concentrations at the target site (Fowler et al. 2020; Gray et al. 2013). However, even with local delivery, AAVs and LNPs remain limited by their immunogenicity, poor uptake by target cells and activation of the humoral immune response, ultimately preventing repeated administration (Barua and Mitragotri 2014; Bulcha et al. 2021; Hargrove et al. 2008; Kuranda et al. 2018; Lee et al. 2023; Maes et al. 2019; Nisini et al. 2018). These limitations underscore the need for alternative, non‐viral gene delivery platforms with improved biocompatibility, enhanced CNS targeting and the ability to support redosing. Addressing these challenges requires further exploration of novel delivery vehicles that can overcome the current barriers to effective CNS gene therapy.

In recent years, extracellular vesicles (EVs) have emerged as promising vehicles for delivering bioactive cargo. EVs play a significant role in intercellular communication and possess an intrinsic ability to transport a variety of molecular signals, thereby mediating various physiological functions (Fitzgerald et al. 2018; Pirisinu et al. 2022). As natural drug delivery vehicles, EVs are inherently biocompatible (Fitzgerald et al. 2018; Pirisinu et al. 2022). Despite significant progress in optimising EVs for therapeutic use, challenges persist, including low yield, difficulties in scaling up production and concerns about cost‐effectiveness (Herrmann et al. 2021; Kim et al. 2024). Additionally, the presence of nucleic acids in EVs from various cell sources can complicate their use in therapeutic applications (Herrmann et al. 2021; Kim et al. 2024).

Recent developments have focused on addressing these issues to improve the utility of EVs as drug delivery systems. Our group has harnessed red blood cell‐derived extracellular vesicles (RBCEVs) as vehicles for drug delivery (Nguyen et al. 2023; Usman et al. 2018). RBCEVs offer several advantages including the absence of DNA, the abundant availability of their source cells (RBCs), and an established safety profile in blood transfusions. Using RBCEVs from O‐negative blood further minimises the risk of immune reactions, allowing for repeated dosing (Nguyen et al. 2023; Usman et al. 2018). These advantages, combined with the ability to administer RBCEVs locally for targeted delivery, make them a superior candidate compared to LNPs and EVs derived from other cell types. Our group has previously used RBCEVs to deliver RNA molecules and anti‐sense oligonucleotides (ASOs) to different cell types for the treatment of several diseases (Usman et al. 2018; Chen et al. 2022; Peng et al. 2022; Peng et al. 2023). For example, we have delivered ASOs to target oncogenes in acute myeloid leukaemia cells and breast cancer cells, siRNAs to the muscles for treatment of cancer cachexia, as well as immunomodulatory RNA (immRNA) to cancer cells for treatment of primary and metastatic breast cancer (Usman et al. 2018; Chen et al. 2022; Peng et al. 2022, 2023). RBCEVs loaded with ASOs effectively silenced oncogenes such as miR‐125b and *FLT3 ITD* in cancer cells, leading to reduced tumour growth and improved survival rates (Usman et al. 2018; Chen et al. 2022; Peng et al. 2022, 2023). The delivery of immRNA also activated RIG‐I signalling, resulting in increased production of type I interferon and pro‐inflammatory cytokines, enhanced immune cell infiltration and inhibited tumour growth (Peng et al. 2022). These findings show that RBCEVs can effectively carry nucleic acids, however, systemic administration of RBCEVs led to minimal delivery to the brain (Usman et al. 2018; Chen et al. 2022; Pham et al. 2021).

In this study, we assessed the potential of RBCEVs to transport plasmids to the CNS via intrathecal administration and evaluated their safety in mouse and non‐human primate (NHP) models. We successfully loaded plasmids onto RBCEVs and demonstrated their distribution within the CNS after intrathecal injection in a mouse model. Our data showed that RBCEVs effectively delivered plasmids to the CNS, resulting in the functional expression of both cytosolic and secretory proteins. Furthermore, our safety assessment in both murine and NHP models revealed no observable systemic toxicity or astrogliosis following intrathecal administration of nucleic acid‐loaded RBCEVs. Taken together, our study demonstrates the expression efficacy and safety profile of plasmid‐loaded RBCEVs for potential gene therapy in the CNS.

## 2 Results

### 2.1 Purification and Characterisation of Nucleic Acid‐Loaded RBCEVs

RBCEVs were isolated utilising the method established in our previous study (Pham et al. 2021). The isolated RBCEVs were enriched in the common EV markers ALG‐2‐interacting protein X (ALIX) and tumour susceptibility gene 101 (TSG101), the RBCEV marker glycophorin A (GPA) and the RBC protein haemoglobin A (HBA) (Figure S1a). Additionally, the cytoskeletal protein β‐actin (ACTB) and the nuclear markers Histone H3 (H3) and Lamin B1 (LMNB1) were undetectable in RBCEV samples, indicating that the purification process effectively removed cellular contaminants (Figure S1a). Additionally, cryogenic electron microscopy (cryo‐EM) provided high resolution visualisation of RBCEVs, confirming their structural integrity without any signs of aggregation or membrane rupture (Figure S1b).

For DNA delivery to the nervous system, RBCEVs were loaded with plasmid DNA using REG1. RBCEVs were separated from unbound plasmids using three rounds of centrifugation. DNA loading efficiency was assessed by lysing plasmid‐loaded RBCEVs and quantifying DNA content via gel densitometry. The presence of a DNA band confirmed successful plasmid loading into RBCEVs (Figure S1c). Quantification of the DNA band intensity showed that approximately 80% of the DNA plasmid was loaded into the RBCEVs (Figure S1d). The loading efficiency and DNA copies per RBCEV were calculated using the parameters shown in Tables S2–S3. Additionally, the diameter of plasmid‐loaded RBCEVs (∼163.4 nm) was comparable to that of unloaded RBCEVs (∼159.2 nm), as determined by nanoparticle tracking analysis, indicating that the RBCEV size remained unaffected during the loading process (Figure S1e).

To assess whether the DNA payload is protected, the control plasmid and loaded RBCEVs were subjected to DNase treatment (Figure S1f). In the absence of DNase, both naked plasmid (negative control) and plasmid‐loaded RBCEVs exhibited well‐defined DNA bands, confirming plasmid integrity. DNase‐treated plasmid‐loaded RBCEVs retained approximately 75% of the DNA. However, following DNase treatment, naked plasmid DNA (positive control) was completely degraded. Similarly, lysed plasmid‐loaded RBCEVs showed extensive DNA degradation, suggesting that disruption of the vesicle membrane exposes the plasmid to DNase digestion (Figure S1f). This indicates that DNA payloads are protected in RBCEVs, highlighting the suitability of RBCEVs as a delivery vehicle for plasmids.

To confirm that the transgene expression observed in the cells is mediated by RBCEVs without any interference from free floating REG1‐DNA molecules, we included a REG1‐DNA control and subjected it to the same loading and washing steps as RBCEVs. The loaded RBCEVs and REG1‐DNA control were lysed and the DNA amount was quantified by gel densitometry. The presence of DNA bands on the gel demonstrated the successful loading of the plasmid onto RBCEVs (Figure S2a). No DNA bands were observed in the REG1‐DNA control, suggesting that the washing steps effectively removed unbound DNA (Figure S2a).

This was further evaluated by the addition of varying doses of EGFP DNA‐loaded RBCEVs or similar volumes of the REG1‐EGFP DNA control to HEK‐293T and N2a cells. Flow cytometry performed after 24 h demonstrated dose‐dependent GFP expression in both cell lines, with higher levels of GFP expression observed with increasing amounts of loaded RBCEVs (Figure S2b–c). In contrast, treatment with the REG1‐EGFP DNA control did not result in GFP expression, indicating that the RBCEV loading and washing protocol effectively removed any free REG1 or DNA molecules that may contribute to transfection of cells independent of RBCEVs (Figure S2b–c).

### 2.2 RBCEVs Can Be Delivered Into the Central Nervous System via Intrathecal Administration

**FIGURE 1:**
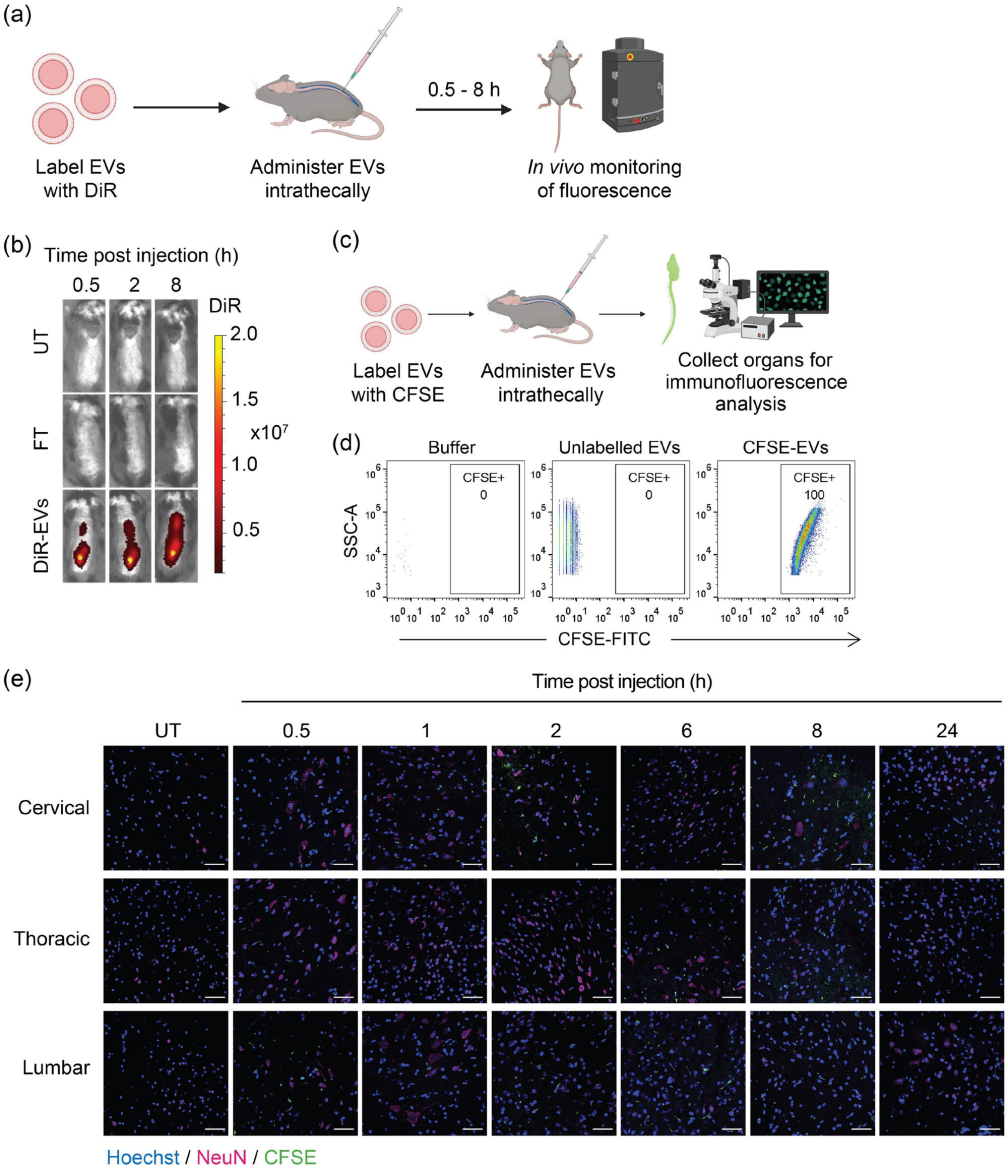
RBCEVs can be uptaken by cells in the CNS after intrathecal administration. (a) Schematic of delivery of DiR‐labelled RBCEVs (100 µg per animal) to the CNS via intrathecal injection in C57BL/6 mice. Mice were imaged over a period of 8 h. (b) Representative DiR fluorescent images of the whole body of mice at different timepoints after intrathecal injections of DiR‐labelled RBCEVs (DiR‐EVs), the flow‐through from the last RBCEV wash (FT), or without receiving any treatment (UT). DiR fluorescence is presented as pseudo‐coloured radiance (p/s/cm^2^/sr). (c) Schematic of CFSE‐labelled RBCEVs (100 µg per animal) delivery to the CNS via intrathecal injections in C57BL/6 mice. The spinal cords were collected at each timepoint for fluorescence imaging. (d) Nano‐flow cytometry analysis to assess the labelling efficiency of CFSE‐labelled RBCEVs. (e) Representative immunofluorescence images of the lumbar, thoracic and cervical regions of the spinal cord at different timepoints after intrathecal injections. Nuclei were stained with Hoechst (blue), neurons with NeuN (pink) and RBCEVs were labelled with CFSE (green). Scale bar, 50 µm (*n* = 3 mice).

The CNS is a challenging target for drug delivery as evidenced by numerous studies (Danon et al. 2019; Gribkoff and Kaczmarek 2017). Systemic delivery of nanoparticles including EVs to the CNS is hindered by the BBB (Wu et al. 2023; Usman et al. 2018; Achar et al. 2021). To demonstrate the potential of RBCEVs for therapeutic delivery for CNS disorder treatment, it is crucial to identify administration techniques that allow RBCEVs to access the brain and spinal cord. To explore this, we first labelled RBCEVs with a lipophilic dye, DiR (DiIC18(7) (1,1′‐Dioctadecyl‐3,3,3′,3′‐Tetramethylindotricarbocyanine Iodide)) and injected them intrathecally into C57BL/6 mice (Figure 1a). DiR‐labelled RBCEVs were washed extensively to remove unbound DiR dye and any resulting micelles. The flow‐through of the last wash was used as a control to determine the background fluorescent level. In mice receiving an intrathecal injection of 100 µg of DiR‐labelled RBCEVs, we observed DiR fluorescence along the spinal cord, which distributed to the brain over the time‐span of 0.5 to 8 h post‐injection, while no signal was detected in the untreated and flow‐through controls (Figures 1b and S3a).

Additionally, to ensure that the DiR signal observed in mice originated from labelled RBCEVs and not from micelles formed by lipophilic dye, a free DiR dye control was processed identically to DiR‐labelled RBCEVs and added to HEK‐293T cells. Flow cytometric analysis revealed that neither the free dye nor the flow‐through controls produced detectable fluorescent signals, confirming that the observed signals were from labelled RBCEVs rather than from DiR micelles formed during the labelling process (Figure S3b).

These findings were further validated by labelling RBCEVs with CFSE dye. Mice were intrathecally administered with CFSE‐labelled RBCEVs and spinal cords were collected at various timepoints post‐injection to monitor EV distribution via immunofluorescence imaging (Figure 1c). Prior to injection, the labelling efficiency of CFSE‐labelled RBCEVs was confirmed by NanoFCM flow cytometry (Figure 1d). Analysis of spinal cord tissues revealed punctate CFSE signals from 0.5 to 8 h post‐injection. The fluorescent signal for CFSE‐labelled RBCEVs were observed in close proximity to nuclei, suggesting cellular uptake (Figure 1e). At 0.5 h timepoint, CFSE signals were primarily localised to the lumbar region of the spinal cord and progressively distributed to distant parts of the spinal cord regions over time. By 24 h, no detectable CFSE signal was observed, indicating clearance of RBCEVs from the CNS within this timeframe (Figure 1e). Taken together, these findings support the potential use of RBCEVs to deliver payloads to the CNS.

### 2.3 RBCEV‐Mediated Delivery of Plasmids Leads to Protein Expression and Secretion in the CNS

**FIGURE 2:**
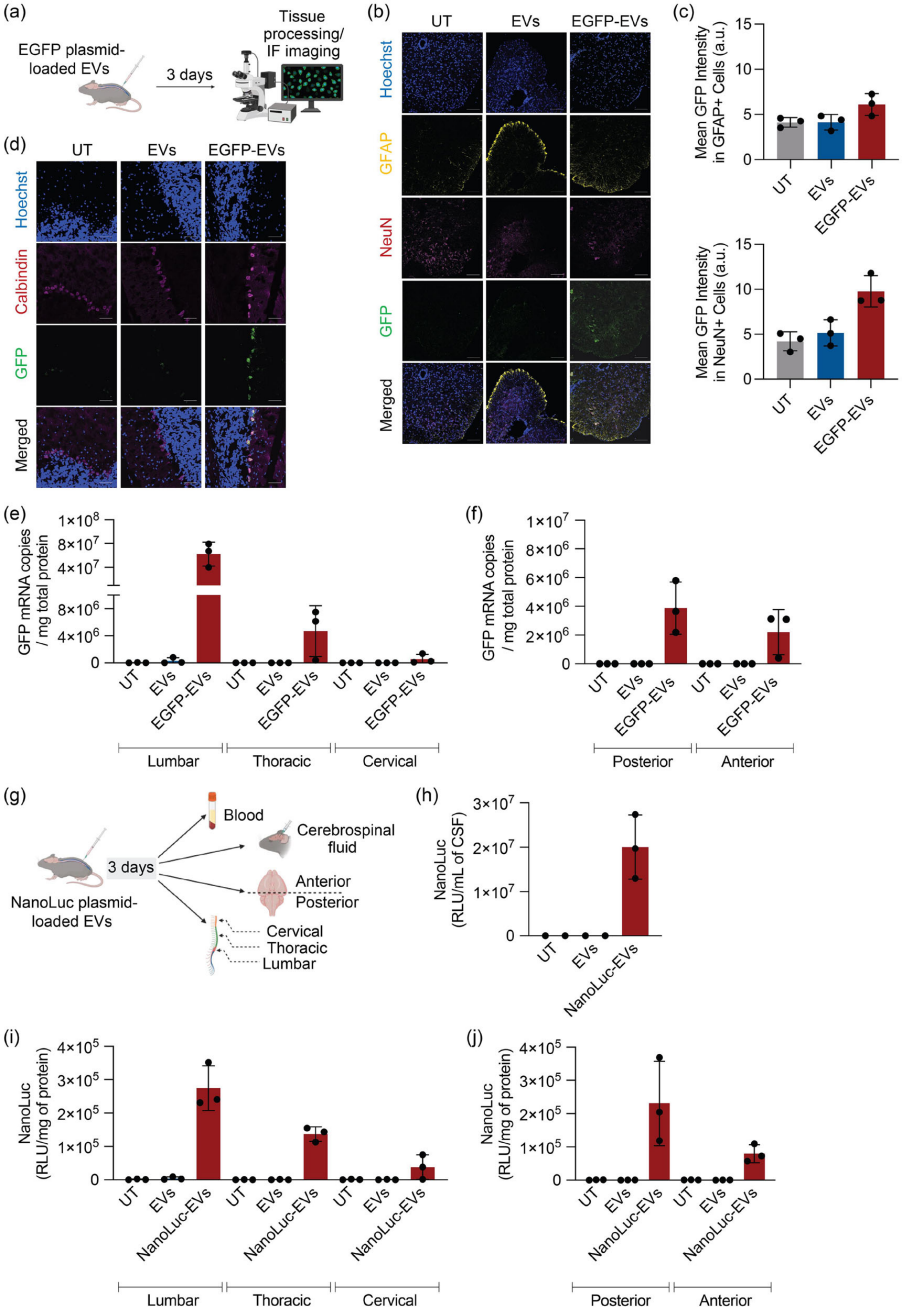
RBCEVs deliver plasmids encoding EGFP and Nano‐Luciferase for protein expression and secretion. (a) Schematic of delivery of EGFP plasmid‐loaded RBCEVs to the CNS via intrathecal injection in C57BL/6 mice. Each treated mouse received 5 × 10^13^ copies/kg of the EGFP plasmid loaded in RBCEVs (equivalent to 1.3 × 10^11^ RBCEV particles or 52 µg RBCEVs per animal) and was sacrificed 3 days post‐injection for sample collection. (b–d) Representative immunofluorescence images of the spinal cord (b), quantification of relative EGFP fluorescence in NeuN+ and GFAP+ cells in the spinal cord (c) and representative images of the cerebellum (d) 3 days after receiving intrathecal injection of EGFP plasmid‐loaded RBCEVs (EGFP‐EVs), RBCEVs only (EVs) or without receiving any treatment (UT). Nuclei were stained with Hoescht (blue). EGFP proteins were stained with anti‐EGFP antibody (green). Cells were stained with the respective cell‐specific markers (NeuN for neurons, GFAP for astrocytes and Calbindin D28k for Purkinje cells). Scale bar, 50 µm. (e–f) qPCR analysis of EGFP mRNA copies in the spinal cord (e) and brain tissues (f) of mice, normalised to total protein, following treatment shown in (a). (g) Schematic of delivery of NanoLuc plasmid‐loaded RBCEVs to the CNS via intrathecal injection in C57BL/6 mice. Each treated mouse received 5 × 10^13^ plasmid copies/kg of NanoLuc plasmid‐loaded RBCEVs (equivalent to 2.17 × 10^11^ RBCEV particles or 87 µg RBCEVs per animal) and were sacrificed three days post‐injection for sample collection. (h–j) Average bioluminescence levels of NanoLuc protein in the cerebrospinal fluid (CSF) (h), tissue lysates of the spinal cord (i) and brain (j) of mice 3 days after receiving intrathecal injection of NanoLuc plasmid‐loaded RBCEVs (NanoLuc‐EVs), RBCEVs only (EVs) or without receiving any treatment (UT). Data are presented as mean ± SD (*n* = 3 mice).

To demonstrate the feasibility of using RBCEVs for the functional delivery of plasmids to the CNS, we loaded RBCEVs with a reporter plasmid encoding the EGFP gene under the control of a CAG promoter and administered them via intrathecal injection in C57BL/6 mice (Figure 2a). To identify the specific CNS cell types that can express the GFP transgene, spinal cord and brain tissues were harvested 3 days after the treatment. Immunofluorescence staining of the spinal cord with cell‐specific markers indicated expression of GFP in neuronal cells (identified by NeuN staining), but not in astrocytes as identified by GFAP staining (Figure 2b,c). In the cerebellum region of the brain, we observed GFP protein expression in Purkinje cells (identified by Calbindin D staining) (Figure 2d). In addition to protein expression, GFP mRNA levels were also quantified in different regions of the spinal cord and brain using qPCR. GFP mRNA was detected in all analysed regions, with the highest levels observed in the lumbar and thoracic area of the spinal cord (Figure 2e,f). These data demonstrate that plasmid‐loaded RBCEVs can deliver DNA cargos to neuronal cells and facilitate the expression of cytoplasmic proteins.

To further strengthen these findings, we assessed the potential of RBCEVs to deliver a transgene that encodes a secreted protein. RBCEVs loaded with CAG‐NanoLuc‐tdTomato plasmid were delivered to C57BL/6 mice by intrathecal injection (Figure 2g). The cerebrospinal fluid (CSF), collected after three days of treatment, showed detectable levels of secreted NanoLuc protein, as determined by NanoLuc assay (Figure 2h). The secreted NanoLuc protein was also found in the serum, suggesting that the NanoLuc protein undergoes bulk flow from the CSF and is effluxed into the bloodstream (Figure S4a). Additionally, we detected NanoLuc activity in the tissue lysates from different regions of the spinal cord and brain. High NanoLuc activity was observed in the lumbar region, followed by the thoracic area and the posterior region of brain (Figure 2i,j). These data further confirm that RBCEVs effectively deliver DNA payloads to distant parts of the CNS, resulting in the expression and secretion of the target protein.

### 2.4 RBCEVs Carrying Plasmids Do Not Cause Systemic Toxicity and Astrogliosis in C57BL/6 Mice Following Intrathecal Delivery

**FIGURE 3:**
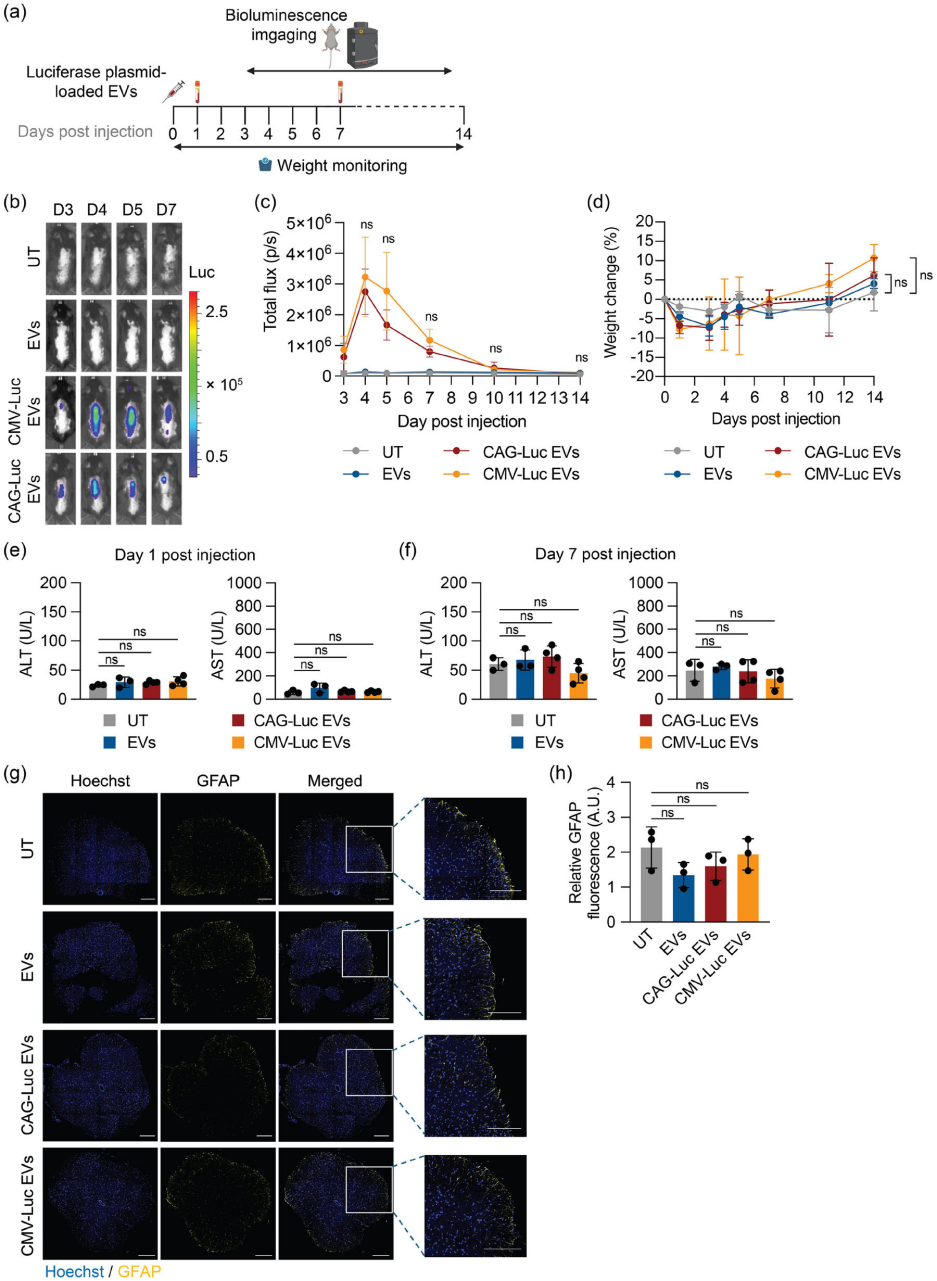
RBCEV‐mediated delivery of luciferase transgene driven by different promoters exhibits similar expression levels and does not cause observable toxicity. (a) Schematic of delivery of luciferase‐encoding plasmid‐loaded RBCEVs to the CNS via intrathecal injection in C57BL/6 mice. Each treated mouse received 5 × 10^13^ copies/kg of luciferase plasmid delivered by RBCEVs (equivalent to 1.7 × 10^11^ RBCEV particles or 68 µg RBCEVs per animal for CAG‐Luc and 1.55 × 10^11^ RBCEV particles or 62 µg RBCEVs per animal for CMV‐Luc) and was monitored for up to 14 days post‐injection. Mice were treated with RBCEVs loaded with either CAG‐luciferase plasmid (CAG‐Luc EVs) or CMV‐luciferase plasmid (CMV‐Luc EVs), or RBCEVs only (EVs), or were left untreated (UT). (b) Representative bioluminescence images of the mice up to 7 days after the treatment shown in (a). Colors indicate bioluminescence signals (photon/s). (c) Quantification of bioluminescence signal in mice shown in (b). (d) Quantification of body weight for up to 14 days after the treatment shown in (a). (e–f) Liver toxicity indicated by the concentration of alanine aminotransferase (ALT) and aspartate aminotransferase (AST) 1 day (e) and 7 days (f) after treatment. (g) Representative immunofluorescence images of the spinal cord 7 days after treatment. Nuclei were stained with Hoechst (blue) and astrocytes with anti‐GFAP antibody (yellow). Scale bar, 200 µm. (h) Quantification of relative GFAP fluorescence. Data are presented as mean ± SD (*n* = 3–5 mice). ns—non‐significant determined by ANOVA with Dunn's or Dunnett's post‐hoc test, comparing CAG‐Luc EVs and CMV‐Luc EVs (c), or compared to untreated (d, e, f and h).

Having demonstrated the functional delivery of plasmids by intrathecal administration of RBCEVs, we then evaluated the distribution of transgene expression and safety profiling of the RBCEV‐mediated DNA delivery platform. To assess this, RBCEVs were loaded with luciferase‐encoding plasmids driven by either the CAG or CMV promoter. Plasmid‐loaded RBCEVs were intrathecally administered to C57BL/6 mice and bioluminescence images were captured over time using in vivo imaging system (IVIS) (Figure 3a). Mice treated with CAG‐ or CMV‐luciferase plasmid‐loaded RBCEVs showed an increase in bioluminescence signal over time, which peaked on day 5 (Figures 3b,c and S4b). No statistically significant difference was observed between the CMV and CAG driven luciferase expression cassettes. A slight decline in body weight was observed across all treated groups, likely due to the stress caused by the intrathecal injection procedure. However, this transient decline in body weight did not reach statistical significance when compared with the untreated group (Figure 3d).

To enable a more comprehensive evaluation of any adverse effects at the protein and cellular levels, blood samples were collected on Days 1 and 7 from mice intrathecally administered with either unloaded RBCEVs, plasmid‐loaded RBCEVs or left untreated. Our data revealed that the intrathecal delivery of both unloaded or plasmid‐loaded RBCEVs did not cause any observable liver or kidney toxicity, as indicated by serum levels of alanine aminotransferase (ALT), aspartate aminotransferase (AST), creatinine, and blood urea nitrogen (BUN) compared to the untreated group (Figures 3e,f and S5a,b). We also evaluated the potential impact of the RBCEV treatment to induce astrogliosis by assessing the intensity of glial fibrillary acidic protein (GFAP) expression using immunofluorescence staining. Our results showed no significant differences in GFAP expression between mice treated with unloaded, plasmid‐loaded RBCEVs, or the untreated group, indicating that the treatment did not induce astrogliosis (Figure 3g,h).

Additionally, the integrity of neuron cells and microglial responses were examined by assessing NeuN and Iba1 expression via immunofluorescence analysis. No significant differences in NeuN or Iba1 intensity were observed between untreated mice and those administered either unloaded or plasmid‐loaded RBCEVs (Figure S5c–f). Histopathological evaluation of spinal cord sections using haematoxylin and eosin (H&E) staining revealed no structural abnormalities, inflammation or tissue damage across all experimental groups, indicating that RBCEV administration did not induce observable morphological pathologies in the spinal cord tissues (Figure S5g). To further evaluate potential neuronal damage, serum neurofilament‐light chain (NF‐L) levels were measured in mice 7 days after intrathecal injection of unloaded, plasmid‐loaded RBCEVs or in untreated controls. ELISA quantification indicated that NF‐L levels in all untreated and treated mice were below the assay's detection limit (data not shown), reinforcing the absence of detectable neuronal injury.

To assess the potential inflammatory response following intrathecal administration of RBCEVs, TNFα and IL‐1β levels were measured in spinal cord lysates 7 days post‐injection of either unloaded or plasmid‐loaded RBCEVs. ELISA quantification revealed that TNFα and IL‐1β levels in both treated and untreated mice were below the assay's detection limit (data not shown). Prior studies have reported TNFα and IL‐1β concentrations as low as 50 and 4 pg/mL, respectively, in healthy mice. (Gajtkó et al. 2020; Obál et al. 2016). These values are close to the sensitivity threshold of the ELISA kit used in this study. Additionally, the positive control provided in the kit confirmed the assay's functionality, demonstrating its ability to detect TNFα and IL‐1β within the expected range. This indicates that RBCEV administration did not induce a measurable inflammatory response in the spinal cord. In conclusion, these findings demonstrate that intrathecal administration of both unloaded and plasmid‐loaded RBCEVs in C57BL/6 mice does not induce overt systemic toxicity, neuroinflammation or histopathological abnormalities in the spinal cord, further supporting the safety profile of this platform.

### 2.5 RBCEVs Carrying Plasmids Do Not Cause Systemic Toxicity and Histopathological Changes in Non‐Human Primates Following Intrathecal Delivery

**FIGURE 4:**
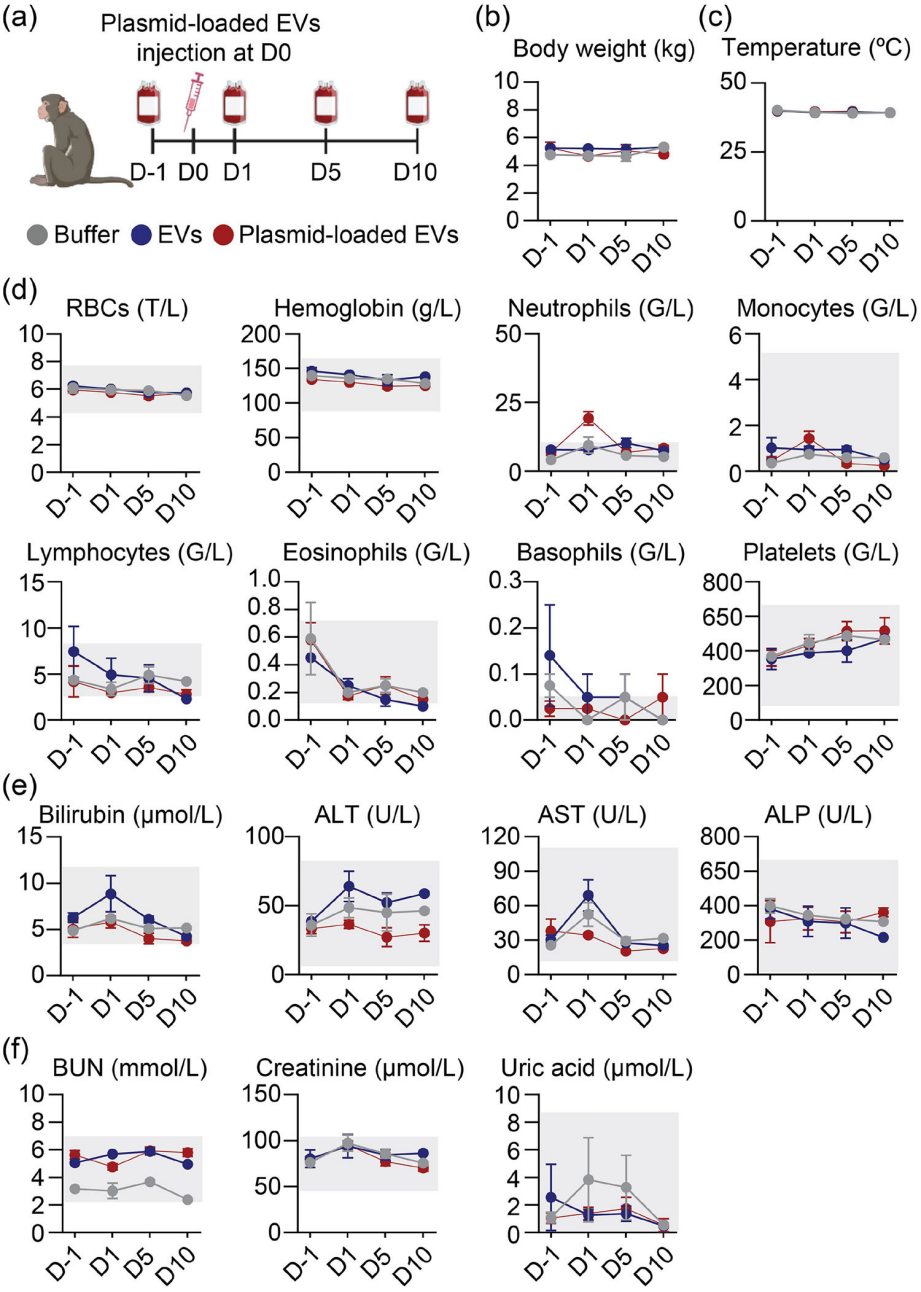
RBCEVs carrying plasmids do not cause observable systemic toxicity in macaques after intrathecal delivery. (a) Schematic of delivery of plasmid‐loaded RBCEVs to the CNS via intrathecal injection in *Macaca mulatta*. Each macaque received 5 × 10^13^ copies/kg of plasmid delivered by RBCEVs (equivalent to 3 × 10^13^ RBCEV particles or 12.5 mg RBCEVs per animal) and was monitored for up to 10 days post‐injection. Treatment groups included plasmid‐loaded RBCEVs, RBCEVs only (RBCEVs) or buffer only (Buffer). (b and c) Quantification of body weight (b) and temperature (c) for up to 10 days after injection. (d) Haematological parameters including concentration of red blood cells (RBCs), haemoglobin (Hb), neutrophils, monocytes, lymphocytes, eosinophils, basophils and platelets. (e) Liver toxicity indicated by the concentration of total bilirubin, alanine aminotransferase (ALT), aspartate aminotransferase (AST) and alkaline phosphatase (ALP). (f) Kidney function indicated by levels of blood urea nitrogen (BUN), creatinine, and uric acid. Data are presented as mean ± SD (*n* = 2–4 macaques). The grey box represents the reference marker values in macaques (d–f). Concentrations are presented in the following units: T/L (× 10^12^ cells/L), g/L (grams/L), G/L (× 10^9^ cells/L), µmol/L (micromoles/L), U/L (units/L) and mmol/L (millimoles/L).

Given the high degree of genetic and physiological similarities between NHPs and humans, NHPs are often considered the gold standard for assessing new drugs (Estes et al. 2018). Additionally, observations made in NHP subjects, including any immune reactions, would provide a better understanding and help anticipate potential responses in human patients. To evaluate the safety profile of RBCEVs, healthy *Macaca mulatta* (commonly known as Rhesus macaques) were intrathecally administered with vehicle, unloaded RBCEVs, or plasmid‐loaded RBCEVs. All animals were closely monitored for any physical and psychological changes in behaviour. Body weight and rectal temperatures were recorded throughout the treatment course. Blood samples were collected at different timepoints to assess blood cell counts and metabolic parameters (Figure 4a).

**FIGURE 5:**
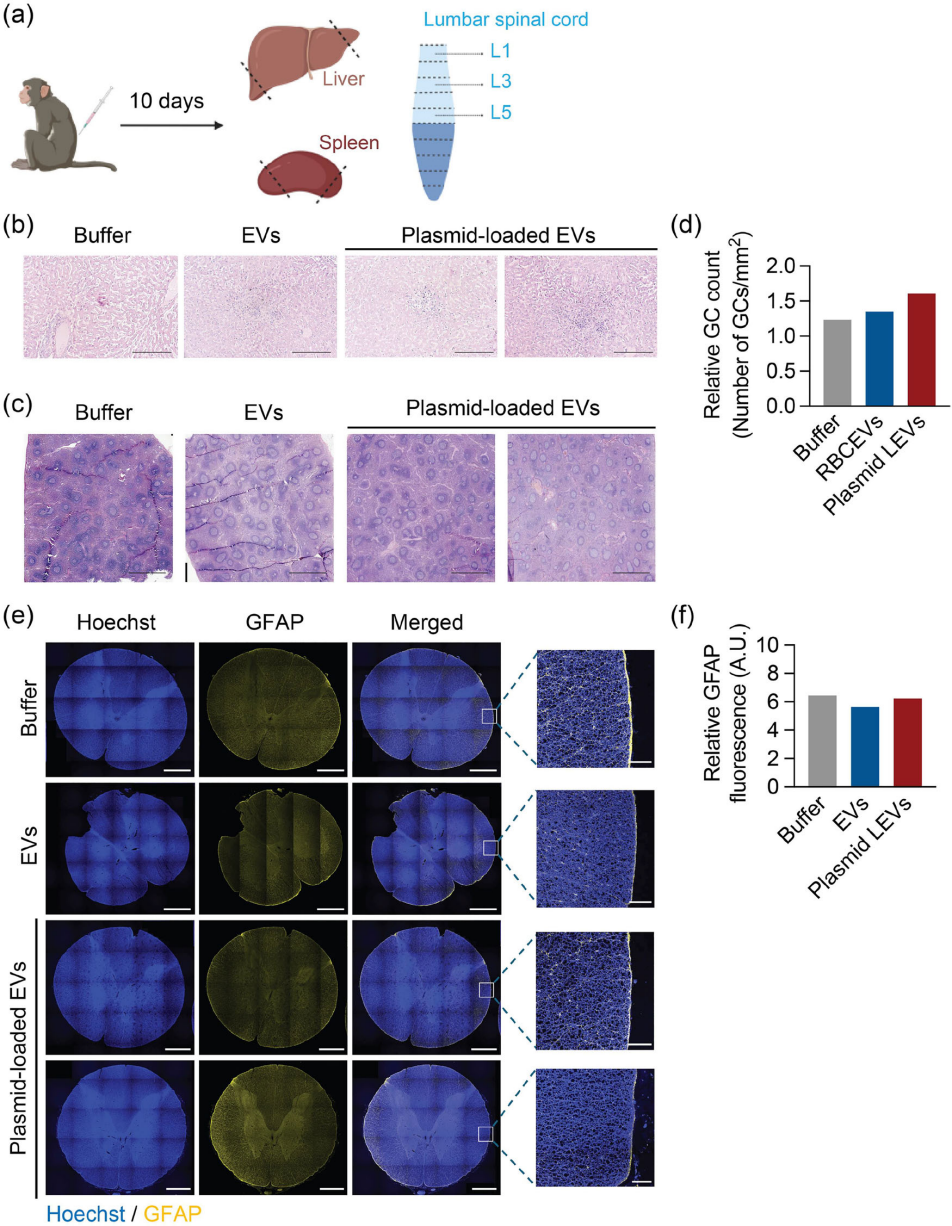
RBCEVs carrying plasmids do not cause histopathological changes in macaque tissues after intrathecal delivery. (a) Schematic of histological analysis of the liver, spleen and lumbar spinal cord from *Macaca mulatta* 10 days after intrathecal delivery of 5 × 10^13^ plasmid copies/kg of plasmid‐loaded RBCEVs (equivalent to 3.12 × 10^13^ RBCEV particles or 12.5 mg RBCEVs per animal), RBCEVs only (EVs) or buffer only (Buffer). Tissue samples for H&E staining were obtained from various regions of the hepatic and splenic tissues, as indicated by the dashed lines. Different regions of the lumbar spinal cord (L1, L3 and L5) were collected for immunofluorescence analysis. (b) Representative H&E‐stained images of the liver 10 days after intrathecal injection. Scale bar, 250 µm. (c) Representative H&E‐stained images of the spleen 10 days after intrathecal injection. Scale bar, 200 µm. (d) Quantification of relative germinal centres (GC) in different regions of the spleen of each control and treated macaque. (e) Representative immunofluorescence images of the lumbar spinal cord 10 days after intrathecal injection. Nuclei were stained with Hoescht (blue) and astrocytes with GFAP (yellow). Scale bar, merged = 1000 µm; magnified = 100 µm. (f) Quantification of the relative GFAP fluorescence in the lumbar spinal cord region of each treated macaque. Buffer and EVs control (*n* = 1 macaque), plasmid‐loaded EVs (*n* = 2 macaques). Each data point is the average quantification of GFAP staining from three different regions of the lumbar spinal cord (L1, L3 and L5).

Encouragingly, all macaques survived well throughout the experimental period, showing no changes in behaviour and body weight compared to pre‐treatment observations (Figure 4b and Table S4). Body temperature remained within the reference range of 36–40°C after treatment across all groups (Figure 4c). Furthermore, measurements for most parameters assessed, including the complete blood count and biochemical analytes in blood, remained within the normal range following treatment (Figure 4d–f). The only blood parameter exhibiting a transient increase in macaques treated with plasmid‐loaded RBCEVs was the neutrophil count, which returned to baseline levels by Day 5 and remained stable until Day 10 (Figure 4d). Post‐mortem examination of tissues from treated macaques also revealed no signs of immune cell infiltration or structural changes in the liver and spleen as evaluated by counting germinal centres (GCs) (Figure 5a–d). Additionally, the lumbar region of the spinal cord showed no significant increase in astrocyte activity, as indicated by the GFAP staining, compared to control groups (Figure 5e–f). Taken together, these findings suggest that RBCEVs can be safely used for plasmid delivery via the intrathecal route of administration in NHPs.

## 3 Materials and Methods

### 3.1 Purification and Quantification of RBCEVs

Whole blood samples were obtained from O negative blood type individuals with informed consent (Innovative Research, Inc, USA). All experiments involving human blood samples were performed with approved ethical guidelines. RBCs were separated from plasma and white blood cells using centrifugation and leukodepletion filters (Nigale, China). Isolated RBCs, in Nigale buffer (0.2 g/L citric acid, 1.5 g/L sodium citrate, 7.93 g/L glucose, 0.94 g/L sodium dihydrogen phosphate, 0.14 g/L adenine, 4.97 g/L sodium chloride, 14.57 g/L mannitol), were diluted up to three times in phosphate buffer saline (PBS; Thermofisher Scientific, USA) containing 0.1 mg/mL calcium chloride. Diluted RBCs were treated with calcium ionophore (Sigma‐Aldrich, USA) at a final concentration of 10 mM at 37°C with 5% CO_2_ to induce vesiculation. RBCEVs were purified as mentioned in our previous study (Chen et al. 2022). Purified RBCEVs were stored in 4% trehalose (Sigma‐Aldrich) in PBS at −80°C.

Quantity of RBCEVs was determined based on haemoglobin content as it is the major constituent of RBCEVs. The haemoglobin contents of RBCEVs were quantified using Haemoglobin Assay Kit (ab234046, Abcam) according to the manufacturer's protocol.

### 3.2 Fluorescent Labelling of RBCEVs

For CFSE labelling, 1 mg of RBCEVs were incubated with 20 µM CFSE (Thermofisher Scientific) for 3 h at 37°C. Following labelling, RBCEVs were spun down at 18,500 × *g* for 30 min. The resulting pellet was resuspended in PBS at 0.1 mg RBCEV/mL overnight at 4°C. The labelled RBCEV pellet was collected and washed twice at 18,500 × *g* for 30 min using centrifugation at 4°C. CFSE signals were analysed by Flow NanoAnalyzer (NanoFCM, UK).

For DiR labelling, 1 mg of RBCEVs were incubated with DiR (Thermofisher Scientific) at 4 mM final concentration for 15 min at room temperature. The labelled RBCEVs were washed once using centrifugation (18,500 × *g* for 30 min), loaded into a size exclusion chromatography (SEC) column; Izon, New Zealand), and eluted with PBS. The RBCEVs were only collected from SEC fractions 7–9 and were washed twice with PBS by centrifugation at 18,500 × *g* for 30 min. As controls, free DiR dye was processed identically to DiR‐labelled EVs to serve as the free dye control, and the supernatant from the final wash was used as the flow‐through control.

### 3.3 Plasmid Loading of RBCEVs and Quantification

Plasmids were loaded into RBCEVs using the REG1 reagent (Carmine Therapeutics, Singapore) according to a pre‐optimised loading protocol. For RBCEV loading, 7 µg of REG1 was diluted in 50 µL of OptiMEM and mixed with 50 µL of OptiMEM containing 1 µg of nanoplasmid (Aldevron, USA). The REG1‐DNA mixture was briefly vortexed and incubated for 15 min at room temperature. Subsequently, RBCEVs were diluted to a concentration of 0.4 mg/mL in OptiMEM and 50 µL of RBCEVs (equivalent to 20 µg) were added to the REG1‐DNA mixture. For homogeneous loading, RBCEVs were placed on an end‐to‐end shaker with a rotation cycle of 15 rpm/min for 1 h at room temperature. After incubation, the loaded RBCEVs were spun down at 18,500 × *g* for 30 min and the pellet was resuspended in PBS. The RBCEVs were further washed three times by centrifugation at 18,500 × *g* for 30 min to remove any unbound REG1 and DNA. The final RBCEV pellet was resuspended in PBS to the required concentration for each experiment and stored at 4°C until use. For intrathecal administration in mice, the dose was prepared in a final injection volume of 10 µL.

For quantification of DNA in RBCEVs, RBCEVs were lysed with 1% Triton‐X (Sigma‐Aldrich) for 5 min at room temperature and incubated with 20 mg/mL heparin sulphate (Sigma‐Aldrich) for 1 h at 37°C. The lysate was separated on 1% agarose gel, stained with GelRed (Sigma‐Aldrich) electrophoresed along with a serial dilution of plasmids in Tris‐acetate‐EDTA buffer (1^st^ BASE, Singapore) for 30 min at 100 V. The gel was imaged using ChemiDoc XRS+ Imaging System (Bio‐Rad, USA). The DNA band intensity was measured using ImageJ v.1.8.0 and the serial dilution was used to plot a standard curve to determine the amount of DNA loaded on RBCEVs.

From the gel quantification, the DNA concentration (ng/µL) of the RBCEV sample was obtained and multiplied by the total sample volume to calculate the total DNA recovered. Loading efficiency (%) was calculated as follows: Loading efficiency = DNA recovered / Starting DNA amount × 100. RBCEV concentration was measured by haemoglobin assay and multiplied by the total sample volume to determine the total EV yield. The EV recovery (%) was calculated as follows: EV recovery = EV yield / Starting EV amount × 100. The number of plasmid copies per EV particle was estimated based on the plasmid size (Table S2) and EV particle concentration, as measured by NTA (1 µg of RBCEVs = ∼2.5 × 10^9^ RBCEV particles) (Table S3).

RBCEV size distribution and concentration was determined using ZetaView nanoparticle tracking analyser (Particle Metrix, Germany).

### 3.4 Cryogenic Electron Microscopy (cryo‐EM)

Lacey carbon EM grids (EMS) were subjected to a 20 s glow discharge in air using a Harrick Plasma system. Following this, 4 µL of the sample solution was applied to the carbon‐coated side of the EM grid and blotted for 2 s before the grid was flash‐frozen in liquid ethane with a Vitrobot system (Thermo Fisher Scientific). The frozen grids were maintained in liquid nitrogen until imaging. Electron microscopy was conducted using a Tecnai Arctica (Thermo Fisher Scientific) operating at 200 kV and with a Falcon III direct electron detector. Data acquisition was performed manually in low‐dose mode to reduce radiation damage, with images captured at magnifications of 20,000× and 70,000× and a defocus range of 2–4 µm.

### 3.5 DNase Digestion Assay

To assess whether RBCEVs protect their plasmid DNA cargo from enzymatic degradation, a DNase digestion assay was performed. Plasmid DNA was either left unencapsulated (naked plasmid) or loaded onto RBCEVs as previously described. To evaluate the impact of membrane integrity, a subset of plasmid‐loaded RBCEVs were lysed with 1% Triton‐X for 5 min at room temperature before DNase treatment. DNase digestion was carried out using TURBO DNA‐free Kit (Thermo Fisher Scientific) according to the manufacturer's protocol. Briefly, 1 µg of samples based on DNA amount were incubated with 1 unit of DNase I at 37°C for 10 min then inactivated with DNase Inactivation Reagent. Plasmid DNA in RBCEVs was quantified by agarose gel electrophoresis as previously described.

### 3.6 Animals and Intrathecal Procedure

C57BL/6 mice (InVivos, Singapore) at six to eight weeks old were used in the study. Animals were housed in groups of maximum five per cage and maintained in a 12‐h light/dark cycle (lights on and off at 7:00 and 19:00, respectively) in a temperature‐controlled (22–24°C) and humidity‐controlled (45%–55%) facility. Standard chow and water were provided ad libitum. Mice were anesthetised using 1%–3% isoflurane (Baxter, USA). The fur was shaved and sterilised with 70% ethanol. The mice were fixed in position by gripping them tightly by the iliac crest with the thumb and index finger of one hand. The vertebrae space was opened by curving the column along the lowest lumbar spinous process (L6). A total volume of 10 µL of solution was injected using a 31‐gauge Hamilton needle (Hamilton Company, USA) into the L5 and L6 of the lumbar vertebra and the needle was withdrawn 1 min after successful injection. The mice were allowed to wake up and were observed for at least 2 min to ensure that there were no injuries. Buprenorphine (0.1 mg/kg) was given at least once subcutaneously before intrathecal procedure. The mice were sacrificed at the scientific endpoint of the experiment through CO_2_ inhalation and cervical dislocation. The experimental procedures were approved by the Institutional Animal Care and Use Committee (IACUC) at the National University of Singapore.

### 3.7 In Vivo Biodistribution Study of RBCEVs

DiR‐labelled RBCEVs (100 µg per animal) were injected intrathecally in C57BL/6 mice (InVivos). DiR fluorescence was tracked for up to 48 h post intrathecal injection using Lumina II in vivo imaging system (IVIS; PerkinElmer, USA). The mice were kept anesthetised with isoflurane. After 24 h, one group of mice were euthanised, and organs were collected for imaging using IVIS (PerkinElmer). Whole body and tissue fluorescence images acquired were analysed using Living System software (Caliper Life Sciences, USA). The background and autofluorescence were defined using the untreated mice and subtracted from the images using Image‐Math function.

CFSE‐labelled RBCEVs (100 µg per animal) were injected intrathecally in C57BL/6 mice (InVivos). After 2 h, the spinal cord and brain were collected. The tissues were fixed in 10% formalin for 24 h at 4°C and soaked in 15% followed by 30% sucrose before cryo‐embedding.

### 3.8 In Vivo Delivery of Plasmid‐Loaded RBCEVs

EGFP‐encoding nanoplasmid‐loaded RBCEVs (5 × 10^13^ plasmid copies/kg) were injected intrathecally in C57BL/6 mice (InVivos). After 3 days, mice were euthanised, and the spinal cord and brain were collected, the tissues were fixed in 10% formalin for 24 h at 4°C and processed for cryo‐embedding.

NanoLuc‐encoding nanoplasmid‐loaded RBCEVs (5 × 10^13^ plasmid copies/kg) were injected intrathecally in C57BL/6 mice (InVivos). After 3 days, mice were anesthetised with 75 mg ketamine/kg and 1 mg medetomidine/kg. The cisterna magna of the mice was pierced with BD Ultra‐Fine 0.5 mL insulin syringe (BD, USA) for collection of CSF. The blood was collected by cardiac puncture and serum was separated for downstream NanoLuc assay. The spinal cord and brain were collected in tubes with 1.4 mm zirconium oxide beads (Bertin Technologies, USA), and homogenised using Precellys 24 homogenizer (Bertin Technologies). NanoLuc levels were determined using Nano‐Glo Luciferase Assay System (Promega, USA) according to manufacturer's protocol. Luminescence was measured using Spark 10 M (Tecan, Switzerland) multimode plate reader.

### 3.9 In Vivo Delivery of Luciferase Plasmids and Toxicity Assessment

Luciferase‐encoding nanoplasmid‐loaded RBCEVs (5 × 10^13^ plasmid copies/kg) were injected intrathecally in C57BL/6 mice (InVivos). For bioluminescence imaging, mice were anesthetised using Isoflurane (Baxter) and intraperitoneally injected with 150 mg/kg of D‐luciferin (PerkinElmer). The mice were monitored for any signs of distress or change in body weight. Blood was collected by retro‐orbital bleeding on Days 1 and 7 post‐injection. Biochemistry parameters including concentration of aspartate transaminase (AST), alanine transaminase (ALT), creatinine and blood urea nitrogen (BUN) were analysed by Comparative Medicine Diagnostics Lab at the National University of Singapore. After 14 days, mice were euthanised and the spinal cord tissues were collected in 10% formalin and processed for cryo‐embedding and immunofluorescence analysis.

### 3.10 Western Blot

RBCs and RBCEVs were lysed in RIPA buffer supplemented with protease inhibitor. Protein quantification was measured by SMART BCA protein assay kit (iNtRON Biotech, South Korea) according to manufacturer's protocol. Proteins were resolved on 10% SDS‐PAGE gel by gel electrophoresis. Then, proteins were electrotransferred onto nitrocellulose membrane, blocked with 5% skimmed milk (Abcam) in TBS‐T buffer (20 mM Tris, 150 mM NaCl and 0.1% Tween‐20), and incubated with primary antibodies (Table S1) overnight at 4°C. Blots were washed with TBS‐T, incubated in secondary antibody (Table S1) for 1 h, washed with TBS‐T before visualising using WesternBright Sirius HRP substrate (Advansta, USA).

### 3.11 RNA Extraction and RT‐qPCR

Total RNA was extracted from tissue lysates using TRIzol reagent (Thermo Fisher Scientific) and cDNA synthesis was performed using the High‐Capacity cDNA Reverse Transcription Kit (Thermo Fisher Scientific) following the manufacturer's instructions. qPCR was conducted using SsoAdvanced Universal SYBR Green Supermix (Bio‐Rad) to quantify mRNA levels. To determine EGFP mRNA expression, a standard curve was generated using known concentrations of EGFP plasmid. This standard curve was used to calculate the number of plasmid copies from Ct values, which were then normalised to total protein measured by SMART BCA protein assay kit (iNtRON Biotech). EGFP primers were designed as follows: Forward primer 5′‐CGG CCA TGA TAT AGA CGT TGT‐3′ and Reverse primer 5′‐AAG GGC ATC GAC TTC AAG G‐3′.

### 3.12 Non‐Human Primates and Intrathecal Procedure

Eight male rhesus macaques (*M. mulatta*) were used for the study. Animals were housed individually and maintained in a 12‐h light/dark cycle. Standard chow and water were provided ad libitum. Animals weighed between 4 and 6.5 kg prior to dosing.

All experimental procedures were carried out by Polyvac (Hanoi, Vietnam) in accordance with their approved animal protocol. In brief, animals were anesthetised by intramuscular injection of ketamine (6.7 mg/kg; Troylab, Australia). Their hair was shaved and sterilised with two applications of 70% ethanol and one application of iodine. The vertebral column was exposed by curving it along the lowest spinous process (lumbar spinal cord). A total volume of 800 µL of solution was injected using Microstat tuberculin (USA) syringes (≤2% volume error) and 24‐gauge needle at the L4‐L5 region of the lumbar vertebrae. After administration, the needle was slowly removed and the injection site was sterilised with 70% ethanol before returning them to the cages. Animals were closely monitored for any adverse effects and changes in body weight or temperature throughout the study. Blood samples were collected from the femoral vein at different timepoints. Complete blood count and biochemistry parameters analysis was carried out by Center of Disease Control Quang Ninh (Quang Ninh, Vietnam).

### 3.13 Immunofluorescence Staining

For immunofluorescence staining, 4 µm tissue sections were cut using Leica CM3050 S Cryostat (Leica Biosystems, Switzerland). Sections were stained using standard immunofluorescence staining protocol. Briefly, sections were steamed in 0.1 M sodium citrate (pH 6.0) buffer for 30 min and pre‐permeabilised in 0.1% Triton‐X (Sigma‐Aldrich) in PBS (Thermofisher Scientific). Tissues were blocked in 2.5% bovine serum albumin, 2.5% normal donkey serum, 0.1% Triton‐X, 0.1% Tween‐20 in PBS for 1 h at room temperature. Sections were stained with primary and secondary antibodies listed in Table S1 and counterstained with Hoechst (Abcam). Background autofluorescence was quenched using Vector TrueVIEW Autofluorescence Quenching kit (Vector Laboratories) according to manufacturer's protocol and mounted with VECTASHIELD Vibrance Antifade (Vector Laboratories). Images were acquired using FV3000 (Olympus) confocal microscope with FluoView software. Fluorescence was quantified using ImageJ software as total signal intensity (integrated density) normalised to the measured area (ROI).

### 3.14 Histopathology

The NHP liver and spleen tissues were fixed in 10% formalin and processed using Leica TP1020 tissue processor. Briefly, tissues were dehydrated sequentially in 70%, 80%, 90% and 100% ethanol, cleared in three baths of Histo‐Clear, infiltrated with paraffin wax, and embedded in paraffin. For H&E staining, 4 µm tissue sections were cut using Leica RM2255 Microtome and collected on SuperFrost slides (Avantor). The sections were dewaxed in two baths of Histo‐Clear and rehydrated sequentially in 100%, 90% and 70% ethanol. The nuclei were stained with Mayer's haematoxylin, decolorised in 0.1% acid alcohol, and counter‐stained with Eosin Y solution (Abcam). The stained tissue sections were dehydrated in 70%, 90% and 100% ethanol, followed by Histo‐Clear then 100% ethanol, and mounted with VectaMount Permanent mounting media (Vector Laboratories, USA). Images of tissues were acquired using Leica Thunder imaging system (Leica Biosystems, Switzerland).

### 3.15 Statistical Analyses

Student's two‐tailed *t*‐tests were performed using GraphPad Prism 10. One‐way ANOVA, computed using GraphPad Prism 10, was used for statistical analysis among treatment groups with appropriate post‐hoc tests including Tukey's, Dunnett's or Dunn's. *p* values <0.05 were considered statistically significant. Data are presented as the mean ± standard deviation (SD).

## 4 Discussion

Nucleic acid therapeutics have the potential for long‐lasting or even curative effects through gene inhibition, addition, replacement or editing (Kulkarni et al. 2021; Wang et al. 2023). This approach represents a significant advancement over traditional therapies that target proteins, which typically yield transient results (Kulkarni et al. 2021; Wang et al. 2023). However, the potential of nucleic acid‐based therapies to address CNS disorders is hindered by the limitations of current gene delivery methods (Kulkarni et al. 2021; Wang et al. 2023; Barua and Mitragotri 2014). Therefore, exploring alternative platforms for nucleic acid delivery is essential. In this study, we present compelling evidence supporting RBCEVs as a novel and safe nucleic acid delivery system to the CNS.

We first demonstrated that intrathecal administration of RBCEVs results in their accumulation within the CNS (Figure 1). These findings contrast with intraperitoneal administration, which led to minimal RBCEV delivery to the CNS (Usman et al. 2018; Chen et al. 2022). Systemically delivered RBCEVs are rapidly cleared by Kupffer cells in the liver, likely due to the presence of phosphatidylserine on the RBCEV surface, which acts as an ‘eat me’ signal (Pham et al. 2021; Jayasinghe et al. 2021). Therefore, the localised intrathecal route is more effective for CNS delivery, bypassing the intact BBB and avoiding issues related to systemic clearance (Wu et al. 2023; Achar et al. 2021). Our findings suggest that intrathecal administration can overcome these challenges and enable RBCEV accumulation in the CNS.

Having established that RBCEVs can be distributed to the CNS via intrathecal administration, we investigated their capacity to deliver plasmids for transgene expression. RBCEV‐mediated delivery of EGFP plasmid, driven by the CAG promoter resulted in GFP expression predominantly in neurons within the spinal cord (Figure 2b). Interestingly, in the cerebellum region of the brain, GFP was expressed in Purkinje cells rather than NeuN‐stained neurons (Figure 2d). Hence, we showed successful transgene expression in the CNS following intrathecal delivery of RBCEVs loaded with plasmids. However, identifying GFP‐positive cells in other areas of the brain proved challenging, primarily, due to the limitation in injection volume and the accessibility of RBCEVs to cells in the distant parts of the brain following intrathecal injection (Rahman et al. 2023). To address this, future research should explore alternative methods of local administration such as intracisternal injection. Although this method requires surgery and is more invasive, it has been shown to deliver larger volumes and facilitate transgene expression across various brain regions, including the cortex, amygdala, hippocampus, and other areas in mice (Bailey et al. 2020). The potential of intracisternal administration of RBCEVs to achieve broader transgene expression throughout the brain warrants further investigation.

We also explored RBCEVs as carriers for plasmid‐driven protein secretion, given the crucial role of proteins in intercellular communication and their routine clinical use. Recombinant protein therapeutics including antibodies, erythropoietin, insulin, interferon, plasminogen activators, growth factors and colony‐stimulating factors highlight the clinical relevance of secreted proteins (Gupta et al. 2016). The conventional use of purified proteins faces challenges such as high production costs and the need for repeated dosing to achieve sustained therapeutic effects. These challenges could potentially be overcome by using genetically engineered cells to continuously secrete proteins in vivo to amass therapeutic concentrations. (Cheng et al. 2023; Zhu et al. 2017) As a proof‐of‐concept, our study demonstrated that RBCEVs can effectively deliver plasmids encoding secreted NanoLuc protein, leading to detectable protein secretion in CSF, spinal cord and brain tissues (Figure 2h–j). This approach holds promise for gene therapy applications, including vectorised antibody delivery for various diseases. For instance, RBCEVs can be employed for delivery of plasmids encoding antibodies targeting CD133^+^ glioblastoma cancer stem cells (GSCs) for treatment of glioblastoma, or vectorised antibodies targeting tau proteins in Alzheimer's disease and other neurodegenerative diseases (Glumac and LeBeau 2018; Singh et al. 2004; Abskharon et al. 2023; Danis et al. 2022).

To evaluate the safety of plasmid‐loaded RBCEVs, we analysed serum and tissue samples following intrathecal administration in mouse and NHP models. We aimed to determine whether EVs, once cleared by the CSF and released into the blood, exert toxic effects on systemic organs. Previous research indicates that systemically administered EVs are captured by Kupffer cells in the liver, leading to disruption of liver enzymes (Gupta et al. 2020; Kooijmans et al. 2016; Németh et al. 2021). In addition to hepatic clearance through liver metabolism, the kidneys play a critical role in eliminating therapeutic agents via glomerular filtration. Small, unprotected DNA molecules are rapidly cleared by the kidneys, making renal assessment relevant when evaluating systemic toxicity (Masereeuw and Russel 2001; Thompson and Joy 2022). Therefore, we collected blood samples to assess systemic toxicity by evaluating markers indicative of liver and kidney damages. Our results demonstrated that intrathecal delivery of plasmid‐loaded RBCEVs did not lead to damage of the liver or kidney in mice and NHPs (Figure 3, 4, 5). The favourable outcome may be attributed to the localised approach of intrathecal administration, which reduces RBCEV distribution to the peripheral organs. These data indicate that direct delivery of plasmids into the CSF could minimise toxicity by limiting off‐target effects.

In addition, RBCEVs may provide anti‐inflammatory effects that compensate for the inflammatory response associated with recombinant DNA, as observed in both our mouse and NHP experiments. This anti‐inflammatory phenotype may be explained by the delivery of haemoglobin‐derived heme, which has been previously shown to prevent inflammation in peripheral macrophages and in a mouse model of atherosclerosis (Pham et al. 2023).

In conclusion, our research is pioneering in demonstrating for the first time that RBCEVs can be successfully administered into the CNS through the intrathecal route. Our data verified that these vesicles are capable of transporting plasmids, which led to the expression of cytosolic and secreted proteins. This study also represents the first evaluation of RBCEV safety in NHPs, providing crucial insights into their potential for clinical applications. Importantly, this procedure did not induce systemic toxicity or local astrogliosis. Hence, RBCEVs hold significant potential as a non‐viral gene delivery vector for delivering nucleic acids to correct underlying genetic disorders of the CNS.

## Author Contributions

Conceptualization, B.W.S.L., T.T.P., and M.T.N.L.; data curation, B.W.S.L., M.T., and M.T.N.L.; formal analysis, B.W.S.L., M.T., and M.W.; investigation, B.W.S.L., M.T., C.G., T.T.P., H.S., M.B.H.L., T.T.T.N., N.L., L.T.N.T., L.T.N, T.H.N. and M.W.; methodology, B.W.S.L., M.T., and M.W.; validation, B.W.S.L., M.T., C.G., T.T.P., M.B.H.L., T.T.T.N., N.L., L.T.N.T. and M.W.; visualization, B.W.S.L., M.T., M.W., and M.T.N.L.; writing – original draft, B.W.S.L., M.T., A.H.L., and M.T.N.L.; writing – review & editing, B.W.S.L., M.T., A.H.L., M.T.N.L., C.G., T.T.P., H.S., M.B.H.L., T.T.T.N., N.L., and M.W.; funding acquisition, M.T.N.L.; resources, M.T.N.L.; supervision, M.T.N.L; project administration, M.T.N.L.

## Conflicts of Interest

Minh T. N. Le is a scientific co‐founder, and advisor of Carmine Therapeutics, a start‐up company that develops gene therapies. Muhammad Waqas Usman is an employee and shareholder of Carmine Therapeutics. Melissa Tan and Harwin Sidik were employees of Carmine Therapeutics. Other authors declare no competing interests.

## Supporting information




**Supplementary Materials**: jev270116‐sup‐0001‐SuppMat.docx

## Data Availability

The data generated in this study are presented within the article and supplemental information files. Other data are available upon request.

## References

[jev270116-bib-0001] Abskharon, R. , H. Pan , M. R. Sawaya , et al. 2023. “Structure‐Based Design of Nanobodies That Inhibit Seeding of Alzheimer's Patient–Extracted Tau Fibrils.” Proceedings of the National Academy of Sciences 120: e2300258120. 10.1073/pnas.2300258120. PMC10576031 37801475

[jev270116-bib-0002] Achar, A. , R. Myers , and C. Ghosh . 2021. “Drug Delivery Challenges in Brain Disorders Across the Blood‐Brain Barrier: Novel Methods and Future Considerations for Improved Therapy.” Biomedicines 9: 1834. 10.3390/biomedicines9121834. 34944650 PMC8698904

[jev270116-bib-0003] Bailey, R. M. , A. Rozenberg , and S. J. Gray . 2020. “Comparison of High‐Dose Intracisterna Magna and Lumbar Puncture Intrathecal Delivery of AAV9 in Mice to Treat Neuropathies.” Brain Research 1739: 146832. 10.1016/j.brainres.2020.146832. 32289279 PMC7997047

[jev270116-bib-0004] Barua, S. , and S. Mitragotri . 2014. “Challenges Associated With Penetration of Nanoparticles Across Cell and Tissue Barriers: A Review of Current Status and Future Prospects.” Nano Today 9: 223–243. 10.1016/j.nantod.2014.04.008. 25132862 PMC4129396

[jev270116-bib-0005] Bulcha, J. T. , Y. Wang , H. Ma , P. W. L. Tai , and G. Gao . 2021. “Viral Vector Platforms Within the Gene Therapy Landscape.” Signal Transduction and Targeted Therapy 6: 53. 10.1038/s41392-021-00487-6. 33558455 PMC7868676

[jev270116-bib-0006] Chen, H. , M. K. Jayasinghe , E. Y. M. Yeo , et al. 2022. “CD33‐Targeting Extracellular Vesicles Deliver Antisense Oligonucleotides Against FLT3‐ITD and miR‐125b for Specific Treatment of Acute Myeloid Leukaemia.” Cell Proliferation 55: e13255. 10.1111/cpr.13255. 35851970 PMC9436904

[jev270116-bib-0007] Cheng, Q. , L. Farbiak , A. Vaidya , et al. 2023. “In Situ Production and Secretion of Proteins Endow Therapeutic Benefit Against Psoriasiform Dermatitis and Melanoma.” PNAS 120: e2313009120. 10.1073/pnas.2313009120. 38109533 PMC10756300

[jev270116-bib-0008] Danis, C. , E. Dupré , O. Zejneli , et al. 2022. “Inhibition of Tau Seeding by Targeting Tau Nucleation Core Within Neurons With a Single Domain Antibody Fragment.” Molecular Therapy 30: 1484–1499. 10.1016/j.ymthe.2022.01.009. 35007758 PMC9077319

[jev270116-bib-0009] Danon, J. J. , T. A. Reekie , and M. Kassiou . 2019. “Challenges and Opportunities in Central Nervous System Drug Discovery.” Trends in Chemistry 1: 612–624. 10.1016/j.trechm.2019.04.009.

[jev270116-bib-0010] Estes, J. D. , S. W. Wong , and J. M. Brenchley . 2018. “Nonhuman Primate Models of Human Viral Infections.” Nature Reviews Immunology 18: 390–404. 10.1038/s41577-018-0005-7. PMC5970954 29556017

[jev270116-bib-0011] Fitzgerald, W. , M. L. Freeman , M. M. Lederman , E. Vasilieva , R. Romero , and L. Margolis . 2018. “A System of Cytokines Encapsulated in ExtraCellular Vesicles.” Scientific Reports 8: 8973. 10.1038/s41598-018-27190-x. 29895824 PMC5997670

[jev270116-bib-0012] Fowler, M. J. , J. D. Cotter , B. E. Knight , E. M. Sevick‐Muraca , D. I. Sandberg , and R. W. Sirianni . 2020. “Intrathecal Drug Delivery in the Era of Nanomedicine.” Advanced Drug Delivery Reviews 165: 77–95. 10.1016/j.addr.2020.02.006. 32142739 PMC8182643

[jev270116-bib-0013] Gajtkó, A. , E. Bakk , K. Hegedűs , L. Ducza , and K. Holló . 2020. “IL‐1β Induced Cytokine Expression by Spinal Astrocytes Can Play a Role in the Maintenance of Chronic Inflammatory Pain.” Frontiers in Physiology 11: 543331. 33304271 10.3389/fphys.2020.543331 PMC7701125

[jev270116-bib-0014] Glumac, P. M. , and A. M. LeBeau . 2018. “The Role of CD133 in Cancer: A Concise Review.” Clinical and Translational Medicine 7: e18. 10.1186/s40169-018-0198-1. PMC6035906 29984391

[jev270116-bib-0015] Gray, S. J. , S. Nagabhushan Kalburgi , T. J. McCown , and R. Jude Samulski . 2013. “Global CNS Gene Delivery and Evasion of Anti‐AAV‐Neutralizing Antibodies by Intrathecal AAV Administration in Non‐Human Primates.” Gene Therapy 20: 450–459. 10.1038/gt.2012.101. 23303281 PMC3618620

[jev270116-bib-0016] Gribkoff, V. K. , and L. K. Kaczmarek . 2017. “The Need for New Approaches in CNS Drug Discovery: Why Drugs Have Failed, and What Can be Done to Improve Outcomes.” Neuropharmacology 120: 11–19. 10.1016/j.neuropharm.2016.03.021. 26979921 PMC5820030

[jev270116-bib-0017] Gupta, D. , X. Liang , S. Pavlova , et al. 2020. “Quantification of Extracellular Vesicles In Vitro and In Vivo Using Sensitive Bioluminescence Imaging.” Journal of Extracellular Vesicles 9, no. 1: 1800222. 10.1080/20013078.2020.1800222. 32944187 PMC7481830

[jev270116-bib-0018] Gupta, V. , M. Sengupta , J. Prakash , and B. C. Tripathy . 2016. “Production of Recombinant Pharmaceutical Proteins.” Basic and Applied Aspects of Biotechnology 77–101.

[jev270116-bib-0019] Hargrove, P. W. , S. Kepes , H. Hanawa , et al. 2008. “Globin Lentiviral Vector Insertions Can Perturb the Expression of Endogenous Genes in β‐Thalassemic Hematopoietic Cells.” Molecular Therapy 16: 525–533. 10.1038/sj.mt.6300394. 18195719

[jev270116-bib-0020] Herrmann, I. K. , M. J. A. Wood , and G. Fuhrmann . 2021. “Extracellular Vesicles as a Next‐Generation Drug Delivery Platform.” Nature Nanotechnology 16: 748–759. 10.1038/s41565-021-00931-2. 34211166

[jev270116-bib-0021] Jayasinghe, M. K. , M. Tan , B. Peng , et al. 2021. “New Approaches in Extracellular Vesicle Engineering for Improving the Efficacy of Anti‐Cancer Therapies.” Seminars in Cancer Biology 74: 62–78. 10.1016/j.semcancer.2021.02.010. 33609665

[jev270116-bib-0022] Kim, H. I. , J. Park , Y. Zhu , X. Wang , Y. Han , and D. Zhang . 2024. “Recent Advances in Extracellular Vesicles for Therapeutic Cargo Delivery.” Experimental & Molecular Medicine 56: 836–849. 10.1038/s12276-024-01201-6. 38556545 PMC11059217

[jev270116-bib-0023] Kooijmans, S. A. A. , L. A. L. Fliervoet , R. van der Meel , et al. 2016. “PEGylated and Targeted Extracellular Vesicles Display Enhanced Cell Specificity and Circulation Time.” Journal of Controlled Release 224: 77–85. 10.1016/j.jconrel.2016.01.009. 26773767

[jev270116-bib-0024] Kulkarni, J. A. , D. Witzigmann , S. B. Thomson , et al. 2021. “The Current Landscape of Nucleic Acid Therapeutics.” Nature Nanotechnology 16: 630–643. 10.1038/s41565-021-00898-0. 34059811

[jev270116-bib-0025] Kuranda, K. , P. Jean‐Alphonse , C. Leborgne , et al. 2018. “Exposure to Wild‐Type AAV Drives Distinct Capsid Immunity Profiles in Humans.” Journal of Clinical Investigation 128: 5267–5279. 10.1172/jci122372. 30352429 PMC6264647

[jev270116-bib-0026] Lee, Y. , M. Jeong , J. Park , H. Jung , and H. Lee . 2023. “Immunogenicity of Lipid Nanoparticles and Its Impact on the Efficacy of mRNA Vaccines and Therapeutics.” Experimental & Molecular Medicine 55: 2085–2096. 10.1038/s12276-023-01086-x. 37779140 PMC10618257

[jev270116-bib-0027] Maes, M. E. , G. Colombo , R. Schulz , and S. Siegert . 2019. “Targeting Microglia With Lentivirus and AAV: Recent Advances and Remaining Challenges.” Neuroscience Letters 707: 134310. 10.1016/j.neulet.2019.134310. 31158432 PMC6734419

[jev270116-bib-0028] Masereeuw, R. , and F. G. M. Russel . 2001. “Mechanisms and Clinical Implications of Renal Drug Excretion*.” Drug Metabolism Reviews 33: 299–351. 10.1081/DMR-120000654. 11768771

[jev270116-bib-0029] Németh, K. , Z. Varga , D. Lenzinger , et al. 2021. “Extracellular Vesicle Release and Uptake by the Liver Under Normo‐ and Hyperlipidemia.” Cellular and Molecular Life Sciences : CMLS 78, no. 23: 7589–7604. 10.1007/s00018-021-03969-6. 34665280 PMC8629784

[jev270116-bib-0030] Nguyen, P. H. D. , M. K. Jayasinghe , A. H. Le , B. Peng , and M. T. N. Le . 2023. “Advances in Drug Delivery Systems Based on Red Blood Cells and Their Membrane‐Derived Nanoparticles.” ACS Nano 17: 5187–5210. 10.1021/acsnano.2c11965. 36896898

[jev270116-bib-0031] Nisini, R. , N. Poerio , S. Mariotti , F. De Santis , and M. Fraziano . 2018. “The Multirole of Liposomes in Therapy and Prevention of Infectious Diseases.” Frontiers in Immunology 9: 155. 10.3389/fimmu.2018.00155. 29459867 PMC5807682

[jev270116-bib-0032] Obál, I. , G. Klausz , Y. Mándi , M. Deli , L. Siklós , and J. I. Engelhardt . 2016. “Intraperitoneally Administered IgG From Patients With Amyotrophic Lateral Sclerosis or From an Immune‐Mediated Goat Model Increase the Levels of TNF‐α, IL‐6, and IL‐10 in the Spinal Cord and Serum of Mice.” Journal of Neuroinflammation 13: 121. 10.1186/s12974-016-0586-7. 27220674 PMC4879728

[jev270116-bib-0033] Peng, B. , T. M. Nguyen , M. K. Jayasinghe , et al. 2022. “Robust Delivery of RIG‐I Agonists Using Extracellular Vesicles for Anti‐Cancer Immunotherapy.” Journal of Extracellular Vesicles 11: e12187. 10.1002/jev2.12187. 35430766 PMC9013404

[jev270116-bib-0034] Peng, B. , Y. Yang , Z. Wu , et al. 2023. “Red Blood Cell Extracellular Vesicles Deliver Therapeutic siRNAs to Skeletal Muscles for Treatment of Cancer Cachexia.” Molecular Therapy 31: 1418–1436. 10.1016/j.ymthe.2023.03.036. 37016578 PMC10188904

[jev270116-bib-0035] Pham, T. C. , M. K. Jayasinghe , T. T. Pham , et al. 2021. “Covalent Conjugation of Extracellular Vesicles With Peptides and Nanobodies for Targeted Therapeutic Delivery.” Journal of Extracellular Vesicles 10: e12057. 10.1002/jev2.12057. 33643546 PMC7886705

[jev270116-bib-0036] Pham, T. T. , A. H. Le , C. P. Dang , et al. 2023. “Endocytosis of Red Blood Cell Extracellular Vesicles by Macrophages Leads to Cytoplasmic Heme Release and Prevents Foam Cell Formation in Atherosclerosis.” Journal of Extracellular Vesicles 12: e12354. 10.1002/jev2.12354. 37553837 PMC10410060

[jev270116-bib-0037] Pirisinu, M. , T. C. Pham , D. X. Zhang , T. N. Hong , L. T. Nguyen , and M. T. N. Le . 2022. “Extracellular Vesicles as Natural Therapeutic Agents and Innate Drug Delivery Systems for Cancer Treatment: Recent Advances, Current Obstacles, and Challenges for Clinical Translation.” Seminars in Cancer Biology 80: 340–355. 10.1016/j.semcancer.2020.08.007. 32977006

[jev270116-bib-0038] Rahman, M. M. , J. Y. Lee , Y. H. Kim , and C.‐K. Park . 2023. “Epidural and Intrathecal Drug Delivery in Rats and Mice for Experimental Research: Fundamental Concepts, Techniques, Precaution, and Application.” Biomedicines 11: 1413. 10.3390/biomedicines11051413. 37239084 PMC10216595

[jev270116-bib-0039] Rouleau, N. , N. J. Murugan , and D. L. Kaplan . 2023. “Functional Bioengineered Models of the Central Nervous System.” Nature Reviews Bioengineering 1: 252–270. 10.1038/s44222-023-00027-7. PMC9903289 37064657

[jev270116-bib-0040] Singh, S. K. , C. Hawkins , I. D. Clarke , et al. 2004. “Identification of Human Brain Tumour Initiating Cells.” Nature 432: 396–401. 10.1038/nature03128. 15549107

[jev270116-bib-0041] Thompson, L. E. , and M. S. Joy . 2022. “Endogenous Markers of Kidney Function and Renal Drug Clearance Processes of Filtration, Secretion, and Reabsorption.” Current Opinion in Toxicology 31: 100344. 10.1016/j.cotox.2022.03.005. 36777447 PMC9910221

[jev270116-bib-0042] Usman, W. M. , T. C. Pham , Y. Y. Kwok , et al. 2018. “Efficient RNA Drug Delivery Using Red Blood Cell Extracellular Vesicles.” Nature Communications 9: 2359. 10.1038/s41467-018-04791-8. PMC6004015 29907766

[jev270116-bib-0043] Wang, C. , C. Pan , H. Yong , et al. 2023. “Emerging Non‐Viral Vectors for Gene Delivery.” Journal of Nanobiotechnology 21: 272. 10.1186/s12951-023-02044-5. 37592351 PMC10433663

[jev270116-bib-0044] Wu, D. , Q. Chen , X. Chen , F. Han , Z. Chen , and Y. Wang . 2023. “The Blood–Brain Barrier: Structure, Regulation, and Drug Delivery.” Signal Transduction and Targeted Therapy 8: 217. 10.1038/s41392-023-01481-w. 37231000 PMC10212980

[jev270116-bib-0045] Zhu, M. M. , M. Mollet , R. S. Hubert , Y. S. Kyung , and G. G. Zhang . 2017. “Industrial Production of Therapeutic Proteins: Cell Lines, Cell Culture, and Purification.” In Handbook of Industrial Chemistry and Biotechnology 1639–1669. 10.1007/978-3-319-52287-6_29.

