# Supplementary material for: Extracellular Vesicles Administered via Intrathecal Injection Mediate Safe Delivery of Nucleic Acids to the Central Nervous System for Gene Therapy
Source: J Extracell Vesicles. 2025 Jul 7;14(7):e70116. doi: 10.1002/jev2.70116 (PMC12230351; doi:10.1002/jev2.70116)
Supplement: Supplementary file 1 — Supplementary Materials: jev270116‐sup‐0001‐SuppMat.docx [file JEV2-14-e70116-s001.docx]

**SUPPLEMENTARY INFORMATION**


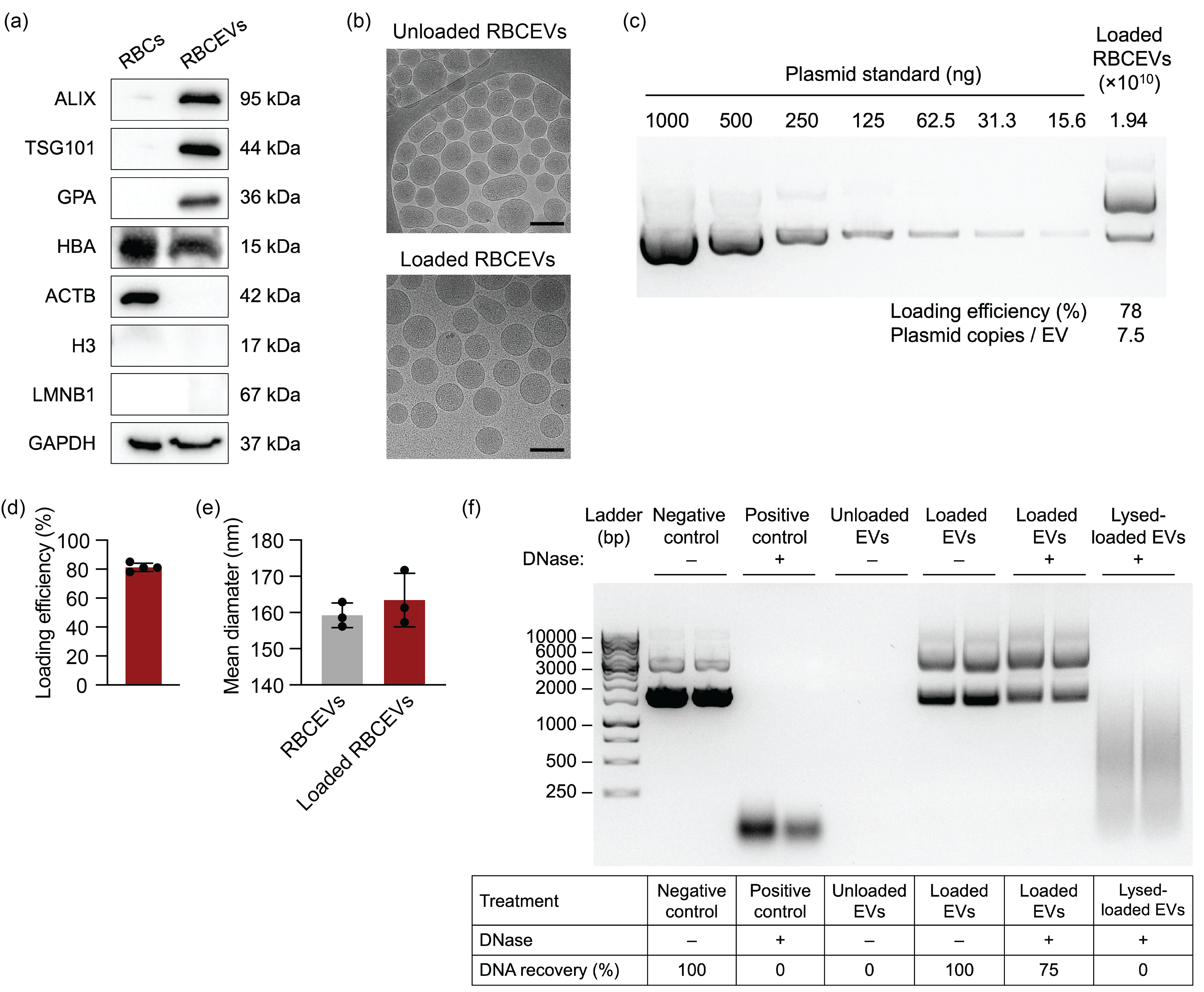


**Figure S1. Characterization of RBCEVs and nucleic acid loaded-RBCEVs. (a)** Western blot analysis of proteins from human RBCs and purified RBCEVs including ALG-2-interacting protein X (ALIX), tumor susceptibility gene 101 (TSG101), glycophorin A (GPA), and RBC protein hemoglobin A (HBA), β-actin (ACTB), Histone H3 (H3), Lamin B1 (LMNB1), and GAPDH. **(b)** Cryogenic electron microscopy image of unloaded and plasmid-loaded RBCEVs. Scale bar, 200 nm. **(c)** Representative agarose gel electrophoresis image of the EGFP plasmid standards and the EGFP plasmid loaded in RBCEVs using REG1 (loaded RBCEVs). **(d)** Average loading efficiency of plasmids in RBCEVs. **(e)** Average diameter of unloaded and EGFP plasmid-loaded RBCEVs (loaded RBCEVs), determined using nanoparticle tracking analysis. **(f)** Agarose gel image following DNase digestion of plasmid-loaded RBCEVs and lysed plasmid-loaded RBCEVs. Lane 1: Ladder; Lane 2-3: 1 µg plasmid without DNase treatment (negative control); Lane 4-5: 1 µg plasmid with DNase treatment (positive control); Lane 6-7: 20 µg unloaded RBCEVs; Lane 8-9: 20 µg loaded RBCEVs without DNase treatment; Lane 10-11: 20 µg loaded RBCEVs with DNase treatment; Lane 12-13: 20 µg loaded RBCEVs (lysed) with DNase treatment. DNA band intensities were quantified and percentage of DNA recovery was calculated by comparing plasmid-loaded RBCEVs with and without DNase treatment.

**
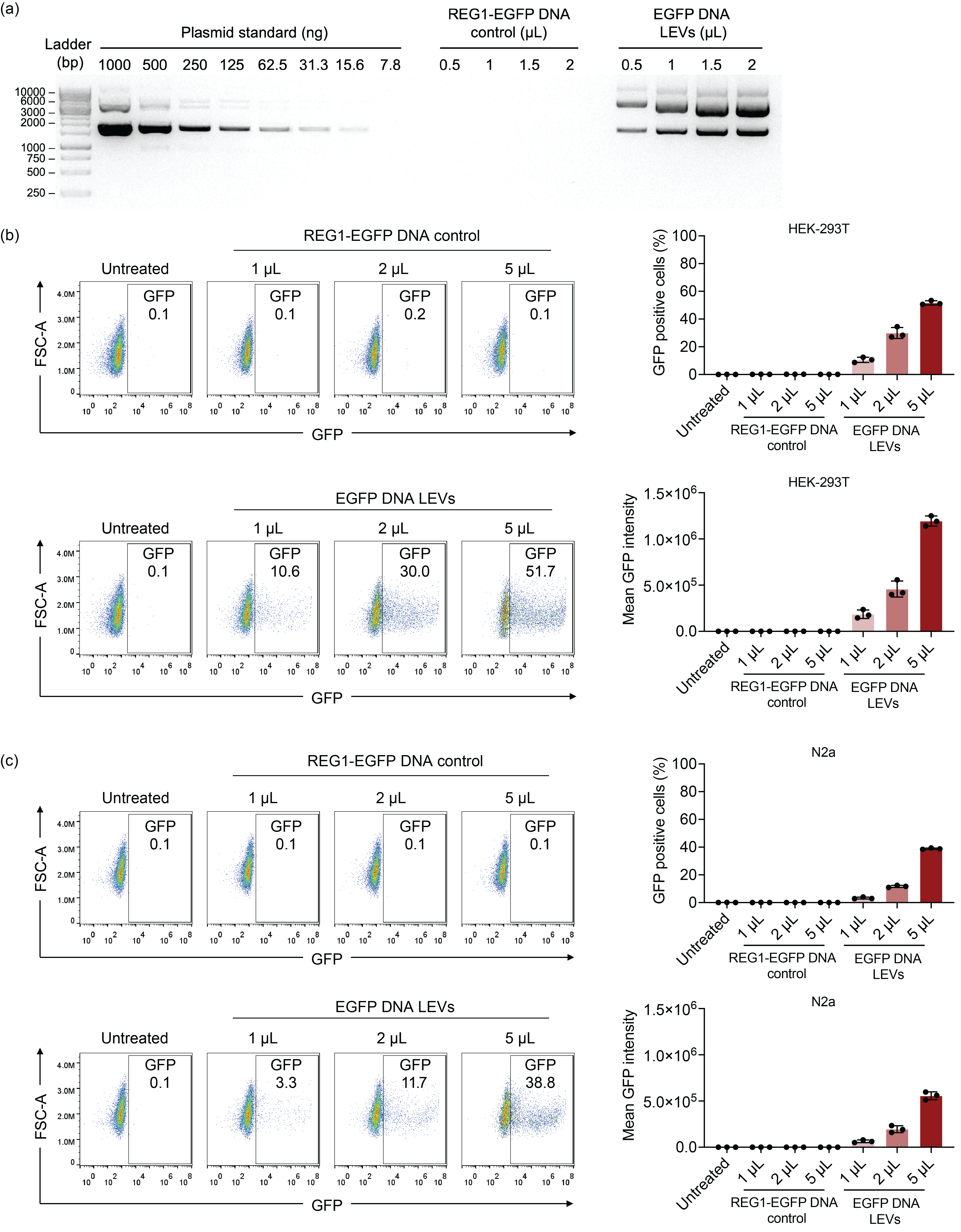
**

**Figure S2. RBCEVs are devoid of free-floating REG1 or DNA after washing. (a)** Representative agarose gel electrophoresis image of the EGFP plasmid DNA standards, the REG1-EGFP DNA control, and the EGFP DNA loaded in RBCEVs using REG1 (EGFP DNA LEVs). **(b-c)** Flow cytometric analysis of HEK-293T (b) and N2a cells (c) 24 h after treatment with the REG1-EGFP DNA control or EGFP DNA-loaded RBCEVs (EGFP DNA LEVs). Data are presented as mean ± SD (n = 3).


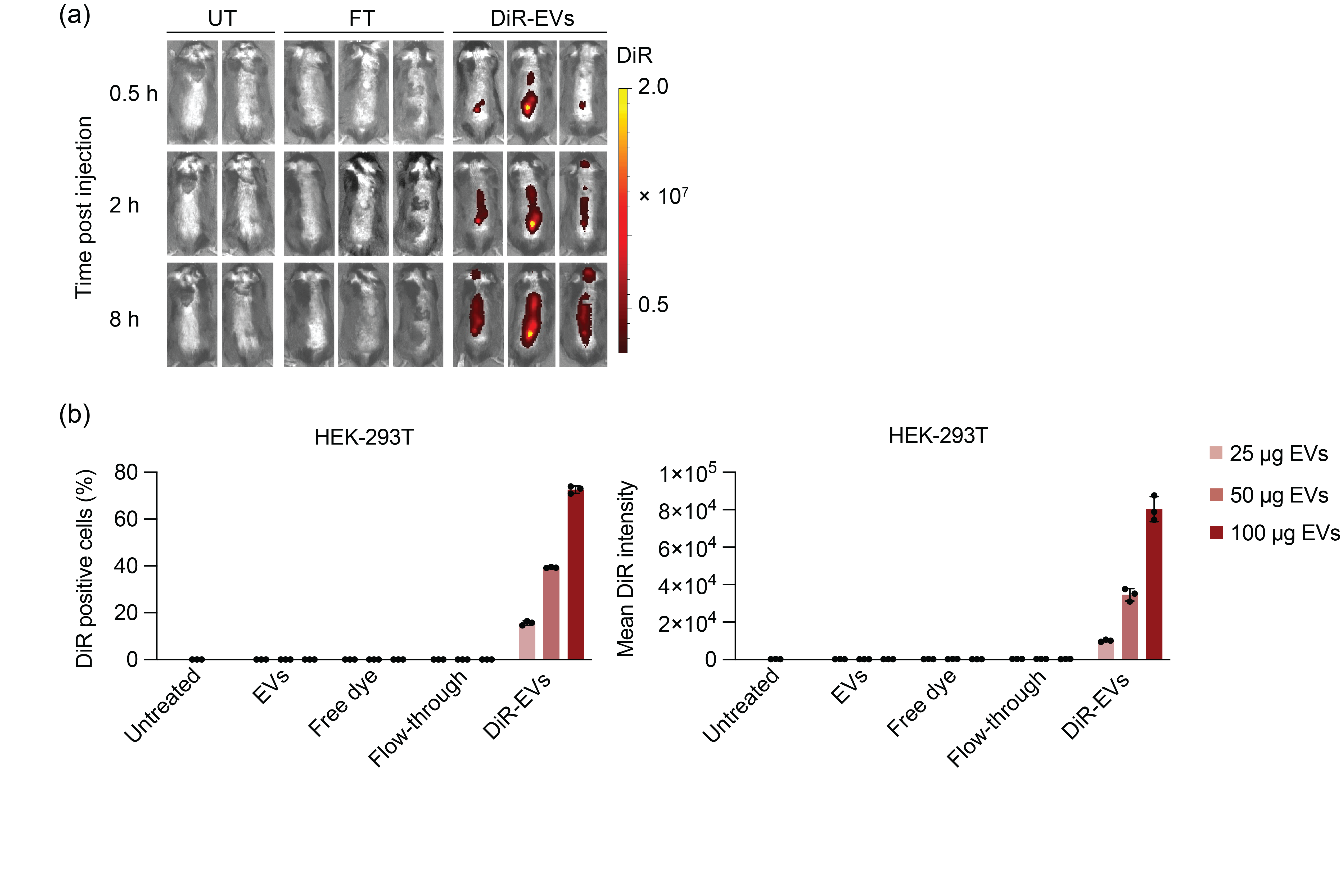


**Figure S3. Uptake of RBCEVs by cells in the CNS after intrathecal administration and validation of DiR labeling specificity. (a)** DiR fluorescent images of the whole body of C57BL/6 mice at different timepoints after intrathecal injections of 100 µg of DiR-labeled RBCEVs (DiR-EVs), the flow-through from the last RBCEV wash (FT), or without receiving any treatment (UT). DiR fluorescence is presented as pseudo-colored radiance (p/sec/cm^2^/sr). **(b)** Flow cytometric analysis of HEK-293T cells treated with the DiR free dye control (processed identically to DiR-labeled RBCEVs), the flow-through from the final wash, and DiR-labeled RBCEVs. Cells were treated with varying doses of RBCEVs and the corresponding volumes for the free dye and flow-through controls. Data are presented as mean ± SD (n = 3).

**
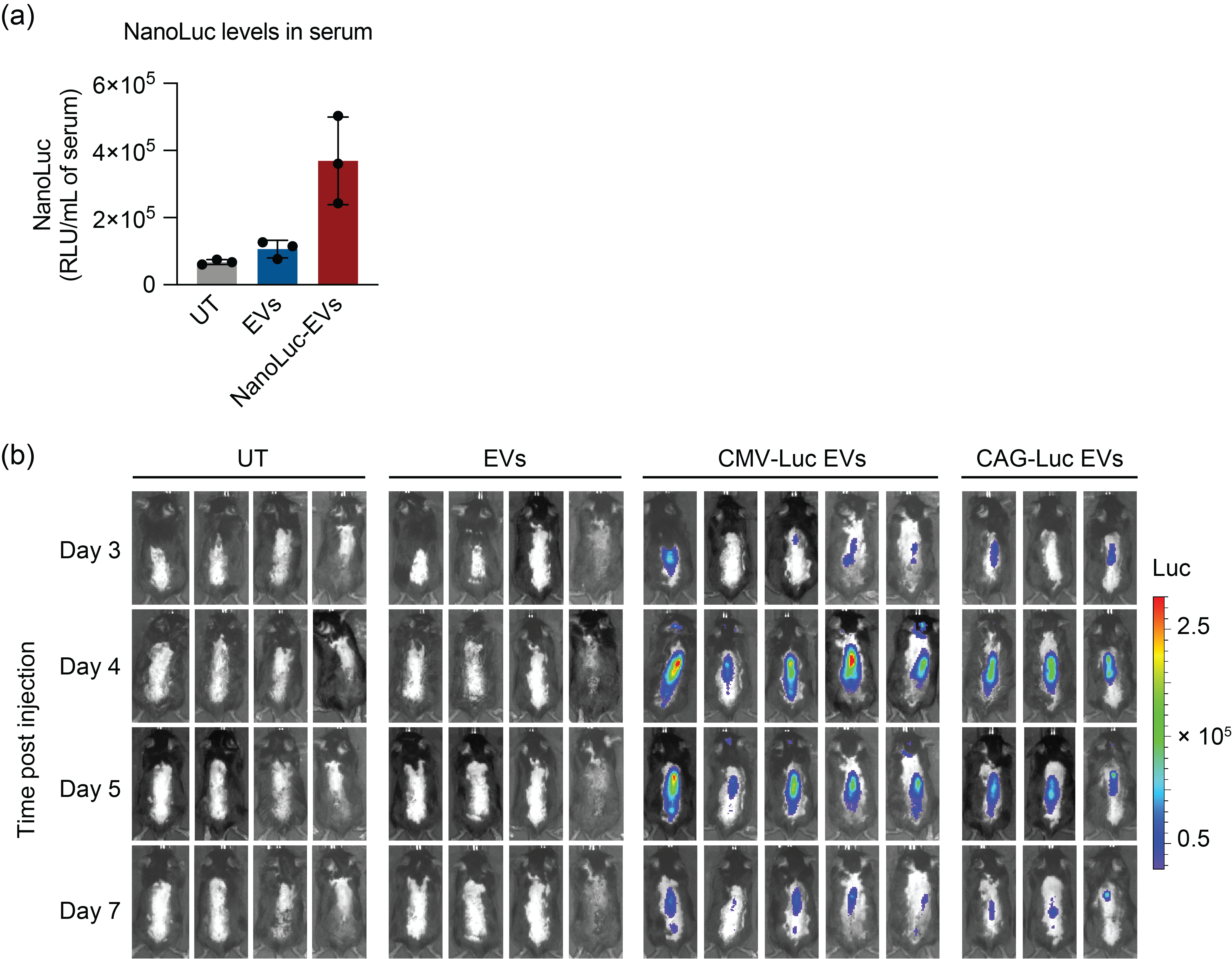
**

**Figure S4. RBCEVs delivering plasmids lead to protein expression and secretion. (a)** Average bioluminescence levels of NanoLuc protein in the serum of C57BL/6 mice three days after receiving intrathecal injection of NanoLuc plasmid-loaded RBCEVs (NanoLuc-RBCEVs), or RBCEVs only (RBCEVs), or without receiving any treatment (UT). Each treated mouse received 5 × 10^13^ plasmid copies/kg of NanoLuc plasmid-loaded RBCEVs (equivalent to 2.17 × 10^11^ RBCEV particles or 87 µg RBCEVs per animal) and were sacrificed three days post-injection for sample collection. Data are presented as mean ± SD (n = 3). **(b)** Bioluminescence images of mice up to 7 days after treatment with intrathecal injection of RBCEVs loaded with either CAG-luciferase plasmid (CAG-Luc EVs) or CMV-luciferase plasmid (CMV-Luc EVs), or RBCEVs only (EVs), or were left untreated (UT). Each mouse received 5 × 10^13^ copies/kg of luciferase plasmid delivered by RBCEVs (equivalent to 3.5 × 10^11^ RBCEV particles or 140 µg RBCEVs per animal for CAG-Luc and 2.56 × 10^11^ RBCEV particles or 103 µg RBCEVs per animal for CMV-Luc). Colors indicate bioluminescence signals (photon/s) (n = 3-5 mice).

**
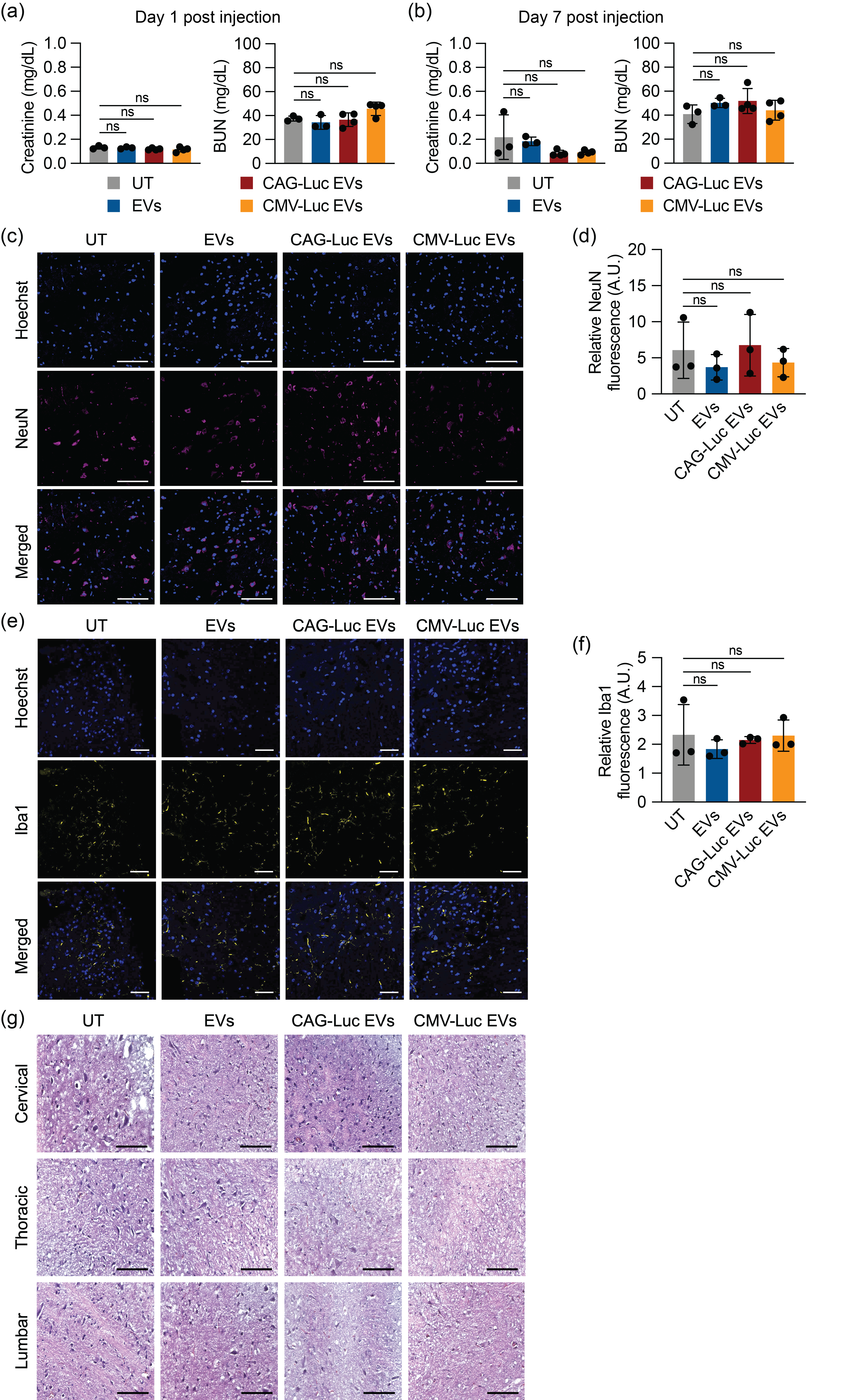
**

**Figure S5. RBCEVs carrying plasmids do not cause observable toxicity in mice after intrathecal delivery.** C57BL/6 mice were treated with intrathecal injection of RBCEVs loaded with either CAG-luciferase plasmid (CAG-Luc EVs) or CMV-luciferase plasmid (CMV-Luc EVs), or RBCEVs only (EVs), or were left untreated (UT). Each mouse received 5 × 10^13^ copies/kg of luciferase plasmid delivered by RBCEVs (equivalent to 3.5 × 10^11^ RBCEV particles or 140 µg RBCEVs per animal for CAG-Luc and 2.56 × 10^11^ RBCEV particles or 103 µg RBCEVs per animal for CMV-Luc). Data are presented as mean ± SD (n = 3-5 mice). **(a-b)** Kidney function indicated by creatinine and blood urea nitrogen (BUN) levels 1 day (a) and 7 days (b) after treatment. **(c-d)** Representative immunofluorescence images (c) and quantification (d) of relative NeuN fluorescence in the spinal cord of mice 7 days after treatment. Nuclei were stained with Hoechst (blue) and neurons with anti-NeuN antibody (pink). Scale bar, 100 µm. **(e-f)** Representative immunofluorescence images (e) and quantification (f) of relative Iba1 fluorescence in the spinal cord of mice 7 days after treatment. Nuclei were stained with Hoechst (blue) and microglia with anti-Iba1 antibody (yellow). Scale bar, 50 µm. **(g)** Representative H & E-stained images of the spinal cord 7 days after treatment. Scale bar, 100 µm. Data are presented as mean ± SD (n = 3 mice).

**Table S1.** List of antibodies used in this study.

| Primary antibodies | | | | |
| --- | --- | --- | --- | --- |
| Name | | Host | Dilution | Product code |
| GFAP | Glial fibrillary acidic protein | Chicken | 1:2000 | NBP1-05198 |
| NeuN | Hexaribonucleotide Binding Protein-3 | Mouse | 1:2000 | 834501 |
| Calbindin | Calcium-binding protein calbindin D-28K | Chicken | 1:5000 | NBP2-50028 |
| Iba-1 | Ionized calcium binding adaptor molecule 1 | Goat | 1:400 | ab5076 |
| GFP | Green fluorescent protein | Rabbit | 1:100 | A-11122 |
|  |  |  |  |  |
| Alix | ALG-2-interacting protein X | Mouse | 1:500 | sc-53538 |
| TSG101 | Tumor susceptibility gene 101 | Mouse | 1:500 | sc-7964 |
| GPA | Glycophorin A (CD235a) | Mouse | 1:2000 | 306602 |
| HBA | Hemoglobin α | Mouse | 1:2000 | sc-514378 |
| ACTB | Beta-actin-HRP | Mouse | 1:5000 | HRP-60008 |
| H3 | Histone H3 | Mouse | 1:1000 | 14269S |
| LMNB1 | Lamin B1 | Rabbit | 1:1000 | ab16048 |
| GAPDH | Glyceraldehyde-3-phosphate dehydrogenase | Mouse | 1:2000 | sc-47724 |
| Secondary antibodies | | | | |
| Name | Dilution | Product Code | | |
| Anti-Mouse-AF488 | 1:1000 | A21202 | | |
| Anti-Rabbit-AF647 | 1:1000 | A31573 | | |
| Anti-Chicken-AF647 | 1:1000 | A21449 | | |
| Anti-Goat-AF647 | 1:1000 | A21447 | | |
| Anti-Mouse-HRP | 1:10000 | 7076P2 | | |
| Anti-Rabbit-HRP | 1:10000 | 7074P2 | | |

**Table S2.** List of plasmids used in this study.

| **Plasmid name** | **Approx. Size (bp)** | **Source** |
| --- | --- | --- |
| CAG-EGFP | 3116 | Aldevron |
| CAG-NanoLuc-tdTomato | 5290 | Aldevron |
| CMV-luciferase | 3949 | Aldevron |
| CAG-luciferase | 5385 | Aldevron |

**Table S3**. Measured values for DNA loading efficiency and RBCEV recovery.

(RBCEVs were loaded with CAG-EGFP plasmid)

|  | Replicate 1 | Replicate 2 | Replicate 3 | Replicate 4 |
| --- | --- | --- | --- | --- |
| EV volume loaded on gel (µL) | 1 | 1 | 1 | 1 |
| DNA concentration (ng/µL) | 467.00 | 495.91 | 499.45 | 532.01 |
| EV amount loaded on gel (µg) | 7.775 | 7.775 | 7.775 | 7.775 |
| EV particles loaded on gel | 1.94e10 | 1.94e10 | 1.94e10 | 1.94e10 |
| Hemoglobin (mg/mL) | 7.775 | 7.775 | 7.775 | 7.775 |
| DNA amount (ng per µg of EVs) | 60.06 | 63.78 | 64.24 | 68.43 |
| Total volume (µL) | 3765 | 3765 | 3765 | 3765 |
| Starting DNA amount (µg) | 2250 | 2250 | 2250 | 2250 |
| DNA recovered (µg) | 1758.25 | 1867.11 | 1880.42 | 2003.00 |
| Loading efficiency (%) * | 78 | 83 | 84 | 89 |
| Starting EV amount (µg) | 45000 | 45000 | 45000 | 45000 |
| EV yield (µg) ** | 29272.88 | 29272.88 | 29272.88 | 29272.88 |
| EV recovery (%) | 65 | 65 | 65 | 65 |
| Plasmid copies / EV particle *** | 7.5 | 8.0 | 8.0 | 8.6 |

* Loading efficiency (%) = DNA recovered / Starting DNA amount × 100

** EV recovery (%) = EV yield / Starting EV amount × 100

*** Plasmid mass was converted to copy number using NEBioCalculator.

**Table S4.** List of selected signs and behaviors used for analyses. (adapted from Joshua J. Smith *et.al.*^1^)

| Cohort #: |  |
| --- | --- |
| Animal ID: |  |
| Day(s) post treatment | |
| Potential life-threatening signs | Does not respond to external stimuli |
|  | Dyspnoea (breathing difficulty) |
|  | Bleeding and/or visible wounds |
|  | Inflammation at site of injection |
|  | Haematuria (bloody urine) |
|  | Melena (blood in stool) |
|  | Watery stool (diarrhoea) |
|  | Weight loss (>10%) |
| Potential signs of clinical issues | Coughing |
|  | Sneezing |
|  | Nasal drainage |
|  | Ocular drainage |
|  | Red and/or swollen eyes |
|  | Emesis (vomiting) |
|  | Hunched posture |
|  | Lying on cage floor (responsive)/lethargic |
|  | Poor coat conditions (i.e matted, ruffled, ungroomed, patchy) |
|  | Pale body coloration |
|  | Dark urine (orange to brown) |
|  | Increased urine output |
|  | Decreased urine output |
|  | Mucus in stool |
|  | Excessively dry stool |
|  | Weight loss (<10%) |
|  | Decreased food consumption |
|  | Failure to take treats |
|  | Vocalization response to touch |
| Atypical behaviours | Hunched posture |
|  | Limping or favouring limb |
|  | Guarding limb or body area |
|  | Repetitive licking, biting or scratching limb/body |
|  | Hair plucking/pulling |
|  | Self-biting |
|  | Circling/pacing in cage |
|  | Flipping |
|  | Head bobbing/weaving |
|  | Bouncing/jumping in place |
|  | Body rocking |
|  | Saluting or eye poking |
|  | Spending time at the back of the cage |
|  | Facing rear of cage |
|  | Failure to groom/engage partner |
|  | Failure to vocalize or engage caretakers |
|  | Excessive vocalization |
| Any other comments |  |

^1^ Smith, Joshua J., et al. "Objective measures of health and well‐being in laboratory rhesus monkeys (Macaca mulatta)." *Journal of medical primatology* 35.6 (2006): 388-396.
